# A Novel Integrated Workflow for Isolation Solvent Selection Using Prediction and Modeling

**DOI:** 10.1021/acs.oprd.0c00532

**Published:** 2021-05-05

**Authors:** Sara Ottoboni, Bruce Wareham, Antony Vassileiou, Murray Robertson, Cameron J. Brown, Blair Johnston, Chris J. Price

**Affiliations:** † EPSRC Centre for Innovative Manufacturing in Continuous Manufacturing and Crystallisation, University of Strathclyde, 99 George Street, Glasgow G1 1RD, U.K.; ‡ Department of Chemical and Process Engineering, University of Strathclyde, 75 Montrose Street, Glasgow G1 1XL, U.K.; § Strathclyde Institute of Pharmacy & Biomedical Science (SIPBS), University of Strathclyde, 99 George Street, Glasgow G1 1RD, U.K.; ∥ Strathclyde Institute of Pharmacy & Biomedical Science (SIPBS), University of Strathclyde, 161 Cathedral Street, Glasgow G4 0RE, U.K.; ⊥ National Physical Laboratory, Hampton Road, Teddington, Middlesex TW11 0LW, U.K.

**Keywords:** solvent selection, purification, solubility prediction, workflow procedure, crystallization, isolation, filtration, washing, drying

## Abstract

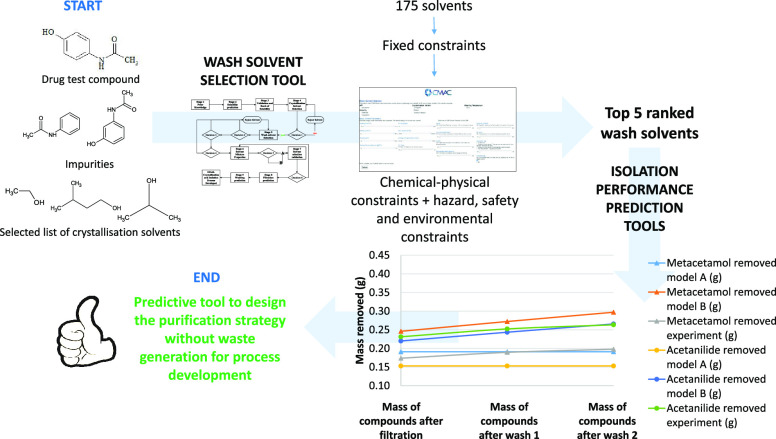

A predictive tool was developed to aid process design and to rationally select optimal solvents for isolation of active pharmaceutical ingredients. The objective was to minimize the experimental work required to design a purification process by (i) starting from a rationally selected crystallization solvent based on maximizing yield and minimizing solvent consumption (with the constraint of maintaining a suspension density which allows crystal suspension); (ii) for the crystallization solvent identified from step 1, a list of potential isolation solvents (selected based on a series of constraints) is ranked, based on thermodynamic consideration of yield and predicted purity using a mass balance model; and (iii) the most promising of the predicted combinations is verified experimentally, and the process conditions are adjusted to maximize impurity removal and maximize yield, taking into account mass transport and kinetic considerations. Here, we present a solvent selection workflow based on logical solvent ranking supported by solubility predictions, coupled with digital tools to transfer material property information between operations to predict the optimal purification strategy. This approach addresses isolation, preserving the particle attributes generated during crystallization, taking account of the risks of product precipitation and particle dissolution during washing, and the selection of solvents, which are favorable for drying.

## 1 Introduction

In recent years, the pharmaceutical industry has faced growing pressure from legislators to develop processes with reduced solvent consumption and waste production and to minimize the use of chemicals, which are toxic to humans and harm the environment.^1^ A recent survey highlighted that the pharmaceutical manufacturing sector has one of the highest waste production rates per kg of product generated. Around 80% of this waste is contaminated solvents.^2,3^ The industry has begun to eliminate the least desirable solvents from active pharmaceutical ingredient (API) manufacturing. One widely used approach is ranking solvents according to environmental risk (e.g., Chem21 and the ACS green chemistry guidelines, and other tools developed by pharmaceutical companies, e.g., GSK and Sanofi guidelines).^4−8^ In addition, the pharmaceutical sector has embraced process intensification approaches, transitioning from batch to continuous manufacturing.^9^ Furthermore, with the trend toward more personalized medicines and lower production volumes, there is an increasing challenge to reduce the quantity of material consumed during process development.

The ultimate goal of the approach developed here is to establish an end to end logical approach supported by predictive and modeling tools to rationally select optimal solvents for isolation of APIs based on the input of crystallization solvent and a limited number of widely available material attributes. The aim of this work is to minimize the amount of experimental work required to design a purification process by

• ranking wash solvents with respect to their interactions with the mother liquor;
- predicting the isolation performance using a mass balance model;
- confirm the recommended isolation solvent using a reduced number of experiments that also validate the conditions to maximize impurity removal and minimize yield loss.

Digital design of continuous API manufacturing offers a path to achieving these goals in an efficient, pragmatic, and time-conscious way. Several predictive tools have been developed to optimize single as well as integrated primary and secondary unit operations, focusing on mass balance, process thermodynamics, and kinetics through the process or process parameters.^10−15^ In the last few decades, researchers have developed solubility prediction tools to identify suitable solvents for synthesis and crystallization;^16−20^ however, little attention has been paid to a solvent selection strategy capable of integrating the entire purification process of crystallization and isolation. The aforementioned methods can be described as “simplistic” tools capable to categorize crystallization solvents based on maximizing yield and crystal purity. However, there is currently no equivalent tool that addresses purification solvent selection, which takes account of other crucial aspects related to particle attributes, process and environmental safety, process parameter ranges, and system operability. Furthermore, the development of an integrated solvent selection approach addressing both crystallization and isolation is novel and will play a crucial part in reducing the issues encountered during isolation (agglomeration, lumping, impurity precipitation, fine particle precipitation, etc.). Crystallization and isolation are intimately connected processes, and decisions taken during crystallization development have a very strong influence on the performance of the downstream isolation process and ultimately on the overall quality of the product crystals.

Typically, the critical quality attributes (CQAs), which must be established during API crystallization and isolation, are purity, particle size distribution (PSD), polymorph, and particle shape. Other attributes may be considered critical in particular circumstances, for example, surface area and roughness. The product crystal size distribution, crystal purity, and polymorphic form are established during crystallization, as a solid suspension in impure mother liquors.^21,22^ The isolation steps of filtration, washing, and drying are necessary to isolate the API maintaining these attributes and must therefore accomplish this without breaking or granulating the crystals or precipitating dissolved product and/or impurities onto crystal surfaces. In order to develop an effective purification strategy, it is necessary to consider the different processing steps to identify conditions that allow production of dry free flowing API with the required PSD while simultaneously meeting the purity requirements consistent with use as a drug substance (i.e., compliant with regulatory guidance, e.g., ICH).^23^

Solvent selection significantly affects the efficacy and operation of each of the individual processing steps. For instance, the crystallization solvent selection must take in consideration the downstream isolation process. Changing the solvent between the crystallization and wash steps is a frequently used strategy in the pharmaceutical industry, for example, switching to a more volatile solvent in which the API with low solubility aids drying. This procedure in particular requires careful design to minimize the formation of particle agglomerates^24^ as further processing is then required to disrupt the agglomerates to retain the desired crystal size distribution. Typically, this is accomplished by milling,^25,26^ which increases process complexity and can negatively affect other particle properties, for example, introducing amorphous character and increasing powder cohesiveness. Washing can dissolve small particles, while this may favorably narrow the PSD at the expense of the isolated yield. The solubility of product in the wash solution would likely lead to agglomeration during drying unless this wash solvent was displaced with another solvent in which the API solubility was significantly lower. According to Murugesan *et al.* and Beckmann,^27,28^ typical industrial practice is to wash a filter cake with at least three cake volumes of solvent, which approximates to between 5 and 7 mL of solvent per gram of API produced. Improving wash efficiency would significantly reduce solvent use and improve environmental metrics.

Here, we present a logical workflow for predictive solvent selection. This includes digital tools to transfer material property information between operations with the goal of selecting the ideal purification strategy. This work enhances the existing solvent selection tools available. This efficient tool can select an API purification process based on maximization of crystallization yield and purity as already seen in previous works^16−19^ with the important additional capability of also minimizing solvent consumption.^29^ Additionally, the preservation of particle attributes, taking account of the risk of precipitation and particle dissolution during washing, and the selection of solvents favoring drying are also considered in this workflow with the goal of global optimization. A series of constrains were selected in accordance with the following assumptions:

- The solvents selected are considered safe and environmentally friendly; the discrimination criteria follow the solvent classifications in the International Harmonisation Guideline ICH6.^23^
- The relative density of crystallization and wash solvent need to be comparable to prevent the risk of solvent layer inversion during washing to avoid cake disturbance reducing wash effectiveness.^27^
- The relative viscosity of crystallization and wash solvent need to be comparable to maximize washing efficiency. Dullien^30^ report that less viscous wash liquors tend to be more effective in entering small capillaries and favor effective solvent displacement and diffusion washing. For this to occur, the wash contact time needs to be long enough to allow for this exchange; however, low viscosity wash solvents tend to pass more rapidly through the cake unless the driving force is reduced to extend the wash duration.
- The thermodynamic properties of the wash solvents (enthalpy of vaporization, boiling point and vapor pressure) need to be selected to favor the downstream drying process. The wash solvent selected should have a low boiling point and enthalpy of vaporization and high vapor pressure to favor the drying process reducing the constant and falling rate drying period.^31^
- Impurities dissolved in crystallization mother liquor should be removed by efficient washing. Impurities already incorporated into the API crystals or precipitated in their own right during the crystallization are not addressed in this workflow. High concentrations of dissolved impurities cannot be fully removed during the isolation process if the impurity solubility is lower than the API solubility: an upper limit for impurity concentration has been chosen to indicate when the efficiency of the wash solvent to purify the cake during washing is likely to be unacceptable.

Any solubility prediction tool can be used to generate the input solubility information required by the workflow. COSMO-RS^32−37^ is one of a range of methods currently available. It should be noted that the accuracy of the quantitative predictions from these models do not currently provide sufficiently accurate quantitative predictions for a wide range of solvent and solutes. However, in the context of this work, we believed that relative qualitative rankings of solvent/solute solubility would suffice as an early indicator to guide laboratory work. In this paper, for example, solubility predictions of chemical entities were generated using the widely used solvent predictive tool COSMO*therm*, which implements the thermodynamic theory of COSMO-RS.^38^ The predicted solubility in a list of wash solvents was experimentally validated. The already selected crystallization solvent was used as the basis to select isolation solvent(s) with chemical and physical properties compatible with the crystallization solvent and compatible with the isolation process. This curated solvent list was then used to predict isolation performance, such as impurity removal, amount of wash solvent to use, propensity of API and/or impurity precipitation during washing, and propensity of API dissolution.

To validate the integrated predictive tool, a series of experiments reported by Ottoboni *et al.*(31) were used to validate the purification solvent selection outcomes. A case study with paracetamol and its related impurities is presented with the aim of meeting a desirable product purity specification and minimizing changes to the crystalline particle attributes occurring during the isolation stage.

## 2 Materials and Methods

### 2.1 Materials

#### 2.1.1 Paracetamol Case Study

Paracetamol (4-acetamidophenol, acetaminophen), Bioxtra, ≥99%, (Sigma Aldrich) and micronized, batch 042213E407, and typical crystalline, batch 637514D001, (Mallinckrodt, Inc.,), acetanilide (99%), and metacetamol (≥99%) (Sigma Aldrich). Absolute ethanol purity ≥99.8% (GC), (Sigma Aldrich), 2-propanol purity ≥99.5% (GC), (Sigma Aldrich), *n*-heptane purity 99%, (Alfa Aesar), *n*-dodecane purity 99%, (Alfa Aesar), and 3-methyl-1-butanol purity 98% (Sigma Aldrich). HPLC was used to determine purity of the isolated product, and the eluents contained water ultrapure, HPLC Grade (Alfa Aesar) and methanol ultrapure, HPLC Grade, 99.8+% (Alfa Aesar). Methanol was also used as the diluent for some samples. Paracetamol shows oral toxicity and skin and eye irritation risks, and it is considered to be a skin sensitizer. Acetanilide is harmful if swallowed. Metacetamol can cause skin, eye, and respiratory irritation.

Ethanol, 2-propanol, *n*-heptane, 3-methyl-1-butanol, and methanol are flammable solvents. Ethanol, 2-propanol, and 3-methyl-1-butanol can cause serious eye damage/irritation. *n*-Heptane, *n*-dodecane, 3-methyl-1-butanol, and methanol can cause skin irritation. 2-Propanol and *n*-heptane can cause drowsiness/dizziness. Methanol is toxic if swallowed. 3-Methyl-1-butanol can cause respiratory damage. *n*-Heptane is very toxic to aquatic life.

### 2.2 Methods

#### 2.2.1 Solubility Prediction Tool

The geometries and polarization charge density for each molecular surface were calculated using COSMO*conf*^39^ and TURBOMOLE.^40^ This allowed molecular parameterization with geometry optimization at the TZVPD-FINE basis set to be applied. Solubility for each solute–solvent combination at 22 **°**C was obtained using the calculated charge densities of the appropriate solute and solvent by the COSMO-RS method implemented within COSMO*therm.*^41,42^

#### 2.2.2 Workflow Procedure

**Figure 1:**
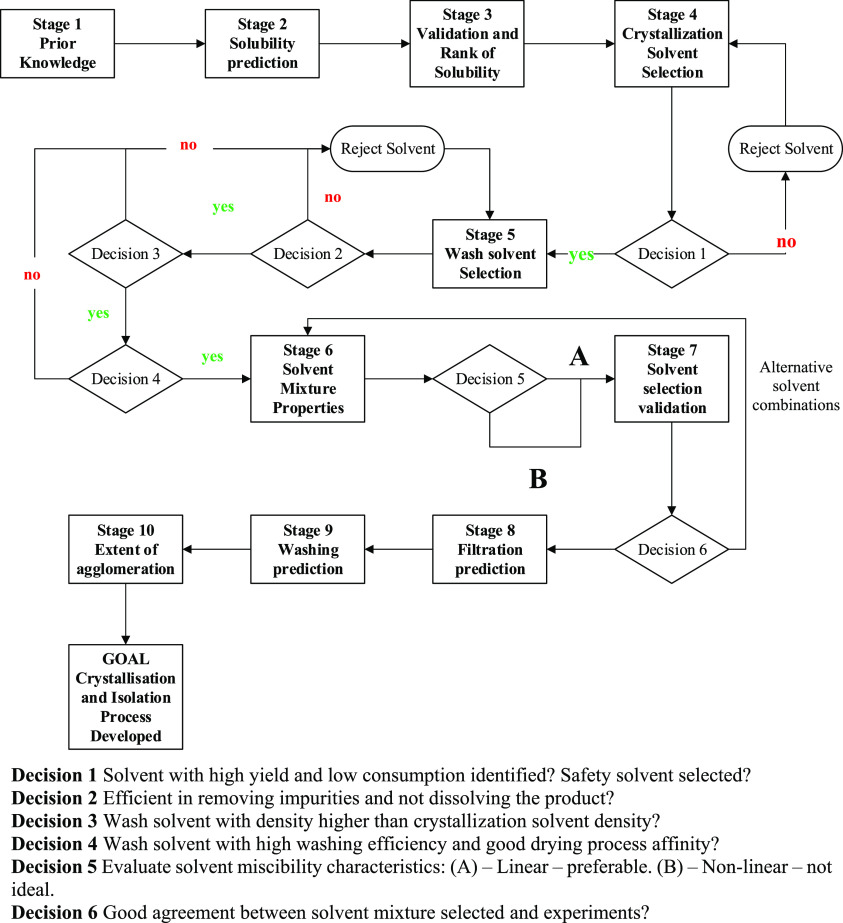
Solvent selection workflow procedure for isolation solvent selection and validation.

The proposed workflow (Figure 1) is divided into nine stages, six of which are related to the selection of crystallization and wash solvent based on the solubility and other relevant solvent properties (e.g., safety, density, viscosity, and thermodynamic properties). The other four stages are related to the isolation performance prediction.

##### 2.2.2.1 Solvent Selection

From stage 1 to stage 4 crystallization solvents are selected and ranked in accordance with the crude stream composition and concentration to achieve high yield and minimize solvent use, as reported elsewhere.^16,19,29^

The ranking criteria selected here maximize the crystallization yield, as reported in Table 1. To maximize yield while minimizing solvent consumption, the ranking order can follow this category order: 1 better than 4, better than 7, better than 2, etc.

**Table 1:**
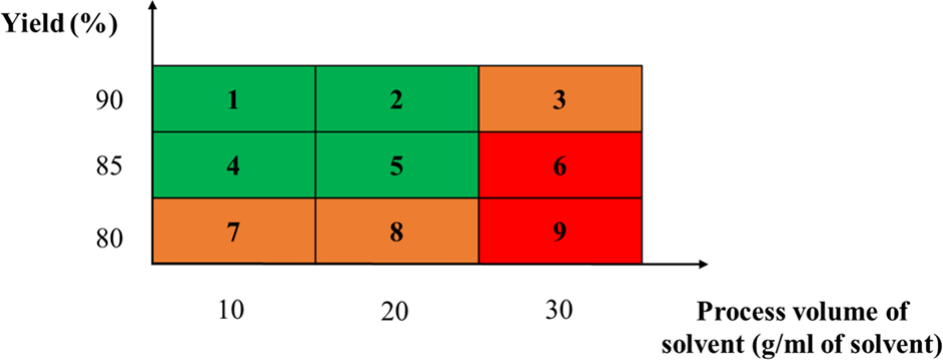
Crystallization Solvent Ranking Criteria Selected^a^

a The yield-process solvent volume ranges considered acceptable to get high isolation yield and good process environmental impact are indicated in green, and the acceptable but not recommended ranges are reported in orange. The ranges of process yield-process solvent volume unlikely to be considered acceptable are reported in red.

From the selected list of solvents, those that pose significant hazardous to humans or the environment (ICH class 1, class 2 can be taken in consideration, even if they are not favorable) are rejected from the list of possible crystallization solvents.

As reported by Jonuzaj *et al.*,^29^ a minimum amount of crystallization solvent is required to achieve a practical crystallization suspension density: if the process volume of solvent is less than 3.5 g solvent/g API, the suspension density of the system is likely to cause mixing issues and the crystallization suspension tends toward a paste. In this workflow, 10 g solvent/g API was set as the preferred maximum suspension concentration.

The selected crystallization solvents are used as inputs to the process to select suitable wash solvents (stage 5). In stage 5, the wash solvents need to pass a series of criteria related to the efficiency of washing, the safety of the solvent, and the compatibility of chemical–physical properties with the crystallization solvent:

a) The efficiency of the wash solvent in removing the impurities without excessively dissolving API is reported in Table 2. Four classifications were designed to remove impurities while minimizing the API dissolution. A good wash solvent shows equal or slightly higher impurity solubility with respect to the API (case 1) at isolation temperature.^43^ In cases where the slurry has a high impurity concentration and lower impurity solubility with respect to the API, these compounds cannot be fully removed during the isolation process. In such cases, it may be necessary to use a large quantity of wash solvent to purify the API (more than 5–6 equivalent cake volumes of wash solvents, or in alternative, if the operator wants to minimize the solvent consumption, the crystallization process needs to be revised). This workflow assumes that >2% as the impurity molar ratio corresponds to high impurity concentration, while less than 2% as the impurity molar ratio is considered as an acceptable amount of impurity to achieve good cake purification during washing in case the delta solubility range is less than 0. In the case of *x* < 0 g/100 g of solvent and low impurity concentration, it is suggested to use at least 3 equivalent cake volumes of wash solvents. The general practice is to use at least 1.5–2 equivalent cake volumes of wash solvents, as reported by Murugesan *et al.*.^27^

b) The safety of the solvent: ICH class 1 solvents are not considered as good candidates and therefore rejected.
c) Density: if the density of the wash solvent is higher than the density of the crystallization solvent (more than 30%^31^), there is the risk of solvent layer inversion causing disturbance to the cake during washing. To prevent this layer inversion, the density of crystallization and wash solvent needs to be comparable or less than that of the crystallization solvent.
d) Viscosity: the viscosity of mother liquor and wash solvent should be similar to promote good displacement washing.^31,43^
e) Thermodynamic properties (boiling point, *T*_b_, enthalpy of vaporization, Δ*H*_vap_, and vapor pressure, *V*_p_): the wash solvent selected should have a low boiling point and enthalpy of vaporization and high vapor pressure to favor the drying process reducing the constant and falling rate drying period;^43,44^ therefore, reducing the ultimate LOD. If the boiling point of the wash solvent is similar to that of the crystallization solvent, there is a risk of particle agglomeration even if the residual mother liquor quantity at the start of drying is low due to enrichment of the less volatile solvent during drying due to API re-dissolution and subsequent recrystallization leading to interparticle bridge formation.^45^ If the practitioners require a more sophisticated approach to identify optimal solvents for drying, using a more refined approach, a VLE model and temperature-dependent solubility could be incorporated into the methodology to account for drying performance.
f) No kinetic effects are considered in this predictive tool.

**Table 2:** Ranking Classification to Select Wash Solvent in Accordance with the Effectiveness in Purifying the Cake while Minimizing API Dissolution^a^

classification	Δ solubility range (g compound/100 g solvent)
1	0 g ≤ *x* < 1
2	1 ≤ *x* < 10
3	10 ≤ *x* < 20
4	*x* ≥ 20

a *x* is the difference of solubility between API and impurity. With the term Δ solubility, the difference between impurity solubility-API solubility is defined.

The role of miscibility of crystallization and wash solvent is evaluated in stage 6 to maximize washing efficiency by promoting displacement, diffusion, and dilution washing mechanisms.^46^ Wash solvent first enters the largest pores in the cake and displaces the filtrate from the connected network of large pores and channels. During displacement washing, there is no capillary pressure equilibrium in the system, but the pressure difference between the two sides of a meniscus at any microscopic point in the system has been assumed to be equal to the capillary pressure as predicted by Laplace’s equation for the continuum. During this process, pressure variation along a sequence of capillaries may be observed.^47^ During the second washing phase, a combination of diffusion and dispersion processes occur.^48^ The filtrate in adjacent network of fine pores held up between crystals may then diffuse into the wash liquid, thus solvent and solute transport occurs due to axial dispersion.

The combination of displacement and diffusion is required to enhance cake purity, so a combination of miscible crystallization and wash solvent is required to form a uniform wash front, as Burisch and Peuker reported.^49^ For this predictive tool, all the crystallization and wash solvents combinations are miscible. The binary plot of the API solubility in the crystallization and wash solvents is predicted with COSMO*therm* to identify the wash curve obtained during washing and the miscibility trend of the two fluids. If the plot shows a maximum, this would result in the risk of API dissolution during the washing process. Presuming the impurity is fully rejected from the crystal lattice during crystallisation, the mother liquor forming the suspension would result in containing the rejected impurities. The selection of crystallization-wash solvent combination presenting a binary plot without maxima favors the elimination of impurities while preventing particle dissolution.^43^

In stage 7, the solvent or solvent mixture selected for washing the filter cake is validated. A design of experiments was used to determine whether the first option of a solvent mixture including the crystallization solvent ranked by the solvent selection tool was the optimal solvent mixture to isolate the selected API. The criteria being preventing impurity precipitation during washing, maximizing the isolated cake purity, and minimizing particle agglomeration. An example of the DoE approach and the characterization techniques used to validate the solvent selection tool are reported elsewhere.^31^

##### 2.2.2.2 Isolation Performances Prediction

To expand the capability of the solvent selection tool, two different modeling approaches were used to estimate the isolation performance by simulating the isolation (filtration and washing) mass balance (stages 8 and 9).

The first modeling tool (model A) considers washing driven by pure displacement, while the second modeling approach (model B) considers washing as a combination of diffusion and axial dispersion washing.

**Figure 2:**
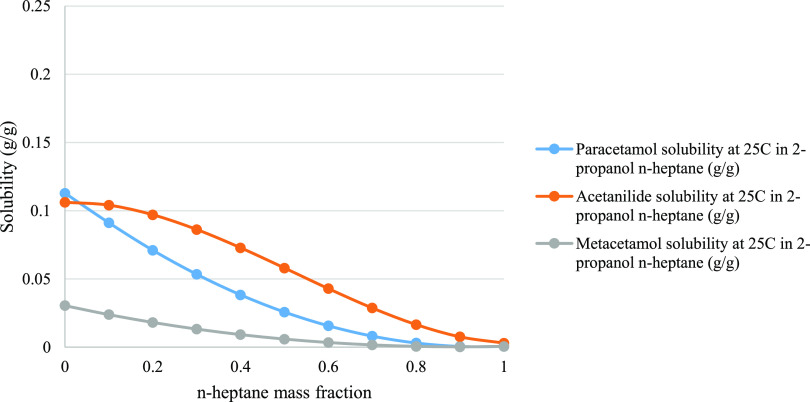
Example of COSMO*therm* binary plot solubility of paracetamol (API), acetanilide, and metacetamol in a gradient crystallization solvent (2-propanol) and wash solvent mixture (*n*-heptane).

Model A simulates an ideal washing process, where a complete mixing of the mother liquor and wash solvent is occurring only when the wash enters the void volume of the cake, while the wash solvent held above the cake is considered not mixed with the mother liquor. To simulate impurity removal and API dissolution the solvent composition in the void volume of the cake follows the simulated solubility gradient (binary plot) of the API and the impurities (Figure 2).

To identify the amount of mother liquor, dissolved API and impurities removed during the filtration prior to washing two approaches are considered: filtration stopped at dryland or filtration stopped at breakthrough^a^. The predictive tool allows the porosity of the cake to be set, and this is used to predict the amount of mother liquor remaining in the cake at the end of filtration (stage 8). Dullien^30^ suggested that the cake porosity of a generic cake is around 30–60% of the total volume. The filtrate and residual composition of the mother liquor in the cake are analyzed in stage 7 to determine the risk of precipitation and the risk of API dissolution. In stage 8, the binary crystallization-wash solvent selected in stage 6 is used to predict impurity removal, the risk of impurity or API precipitation and the risk of API dissolution. Assumptions used in this step are as follows:

- The system simulated is considered as a powder bed of particles with a fixed cake void fraction. Particle properties like, particle size distribution, aspect ratio, and habit are not considered.
- Isolation equipment geometry and size are known and are used in the model, for example, values corresponding to the AWL CFD25 ports were used.
- Filtration is modeled as a simple separation process where two phases are generated; a wet solid phase, corresponding to the cake filtered to dryland (cake saturated to with impure mother liquor), and a liquid phase, corresponding to the filtrate removed. The liquid portions show the same mass fraction of species in solution.
- Washing is considered to be purely driven by a displacement mechanism: no kinetic effects (dilution and diffusion) are considered.
- Cake composition is determined at the end of the washing process.
- Using the solubility binary plot curve of the API and impurities the filtrate composition evolution is predicted considering the evolution of liquid composition from pure crystallization to pure wash solvent, analyzing in increments of 10% of the mass of the wash each time. In this way, this predictive tool discretizes the washing process, considering it as a sequence of 10 time steps during which the liquid phase composition inside the cake change from 100% of mother liquor to a 100% wash solvent. In the first step, 10% of the mass of the wash solvent enters the cake voids and displaces the corresponding amount of the residual mother liquor, and the remaining 90% of the mother liquor then mixes fully with the wash solvent to generate a new mother liquor composition. This process is repeated 10 times until all the wash solvent has entered the cake voids. To determine the risk of API dissolution, the solubility of the API in the fluid composition achieved at the end of each time step is calculated and compared with the dissolved API quantity present at the end of the filtration process. If the quantity of API dissolved after the washing is bigger than the quantity of API dissolved at the end of the filtration process, the risk of API particle dissolution is flagged. To predict the risk of impurity precipitation during washing, a similar approach is taken. For each time step, the predicted solubility of the impurities for a defined fluid composition is calculated. If the quantity of dissolved impurities is higher than their solubility values, there is a risk of impurity precipitation occurring in the filtrate but also in the cake. The residual impurity content for each washing time step is calculated considering the residual impurity content calculated from the previous washing time step.

Model B considers diffusion and dispersion occurring during washing, as modeled by Huhtanen *et al.* and Tien.^50,51^

This model uses the following approximations:

- The cake is formed of polydisperse spherical particles with a known size distribution.
- The system is simulated as a powder bed of particles with a fixed cake void fraction.
- The isolation equipment geometry is known and is used in the model to define the volumes of filter cake and of fluids; in the example data, this corresponds to the size of the AWL CFD25 ports.
- The filter medium resistance is empirically estimated; in the examples, this is set to 1 × 10^+06^ (1/m).
- Filtration is simulated with the process endpoint set to dryland. The mass fraction composition of the liquid phase left in the cake and the filtrate removed is identical as filtration is considered purely as a phase separation process.
- The number of washes and the amount of wash solvent per each wash (expressed in equivalent cake volumes) are selected to simulate the experiments reported in a paper describing the workflow validation.^31^
- The diffusion coefficient selected to model the diffusion washing mechanism was selected to be 1 × 10^–09^ (-), as suggested by Huhtanen *et al.*.^50^

This simulation tool provided predictions of filtration properties, including filtration time, volumetric flow rate, mass fraction of the filtrate, and wet cake composition. The corresponding wash performance results include the washing yield, the washing mass fraction, and the mass of the filtrate removed after each washing step.

#### 2.2.3 Material Characterization

A series of analytical techniques were used to determine the raw material thermodynamic and particle attributes to determine the solubility of the API and its impurities in a series of solvents to validate predicted solubility and to characterize the isolated material.

#### 2.2.4 Solubility

The solubility of paracetamol and related impurities in crystallization and wash solvents was taken from the literature,^52^ where available, compared with predictions using COSMO*therm* (COSMOlogic GmbH & Co. KG, Germany) and confirmed experimentally^31^ by the gravimetric approach using an incubator (Incubator S160D, Stuart, Cole-Parmer, UK) on a multi-position stirrer plate.^43^

#### 2.2.5 Impurity Content during Crystallization and Isolation

The quantity of filtrate removed during each stage of washing was recorded, and the collected fluid was characterized by HPLC to determine the impurity content in the filtrate removed during washing. To quantify the impurity content of the filter cake and filtrate, HPLC calibration curves for pure paracetamol, acetanilide, and metacetamol were gathered using a multilevel calibration method. An Agilent 1260 Infinity II system with a diode array UV detector was used. The column was an Agilent Poroshell 120 EC-C18 4.6 x 100 mm 4 μm operated at 40 °C, with a flow rate of 1 mL/min. The injection volume was 5 μL, data from two wavelengths were used: 243 and 230.5 nm, and the mobile phase was 80% water and 20% methanol.

#### 2.2.6 Particle Size Distribution

Particle size analysis of the different grades of raw paracetamol was carried out using laser diffraction using a Mastersizer 3000 particle size analyzer with an Aero S dispersion unit (Malvern Panalytical). This allows for direct analysis of the dry solid material (method: background measurement duration 10 s, sample measurement duration 10 s, obscuration limit 0.1–15%, stabilization time 0 s, measurement obscuration filtering time out 10 s, feed rate 25%, standard venture dispenser, general purpose tray with hopper, Hopper gap 4 mm). The number of measurements in the sequence was set as 2. The air pressure of the analysis system was set to 0.5 barg.

#### 2.2.7 Filtration and Washing Procedure

Suspensions containing dissolved acetanilide and metacetamol as the representative impurities of synthesis were prepared to a concentration of 2% by mass of each impurity. The required mass of each impurity was weighed and dissolved fully in the crystallization solvent prior to adding any of the paracetamol. The amount of paracetamol required to saturate the solvent solution was then added and dissolved. The final step in the suspension preparation was to add the paracetamol required to form the cake, and this paracetamol represents the solid load, calculated in % by mass. This two-stage addition of paracetamol was crucial to avoid partial dissolution of the cake forming particles affecting their particle size and hence the filter cake properties.

To avoid antisolvent effect^53^ leading to dissolved API being precipitated during the first wash, the first stage wash was prepared using a mixture of pure crystallization and wash solvent. The composition was selected based on the wash solvent screening methodology outlined in the raw materials characterization section 2.2.7. The second washing step was conducted using the pure wash solvent. In each instance, the wash solvent quantity was based on the cake void volume and the criteria set up in the experimental design.

The isolation unit selected to validate the predictive mass balance tool is the CFD25,^31^ a dead end filtration unit designed to filter, wash, and dry API cakes in manual, semi-automated, or continuous mode. A detailed description of the prototype is reported by Ottoboni *et al.*.^31^ The experiments were conducted in the unit’s optimization mode. Details of the experimental and characterization procedures are reported elsewhere.^31^

## 3 Results and Discussion

### 3.1 Solubility Prediction Tool/Predicted Crystallization and Wash Solvent Combinations

A solvent list containing 173 solvents was curated (see the Supporting Information). Key solvent properties required for this work were added to this list where available. The GSK solvent risk classification^4^ scores were also added to the relevant solvents. This list formed the basis for the solvent screening work. Solubility data for the API and impurities in all the solvents were calculated, and subsequently filters and optimization techniques were applied to this data set. Only one filter was applied before running the jobs in COSMO*therm*, this was to remove solvents where their boiling point or melting point was within 10 °C of the washing temperature (22 °C). This reduced the list to 159 solvents (see the Supporting Information). Multi-objective optimization, utilizing the NSGA-II Pareto sorting algorithm, was then applied to these data to minimize paracetamol solubility in the wash solvent while maximizing the solubility of the impurities (see the Supporting Information).^54^ These results did not take in to account the effects of the crystallization solvent (see the Supporting Information). As such, warning flags were then added to the data to allow further filtering. Wash solvents were flagged if the API solubility is more in the wash solvent than the crystallization solvent, density of the wash solvent is more than 1.3 times of crystallization solvent, and when wash solvents were not fully miscible with the crystallization solvent. Miscibility data for all solvent combinations were calculated within COSMO*therm* and verified/corrected where literature data were available. Removing any solvent with a warning flag before performing the multi-objective optimization results in a ranked list for each crystallization solvent. The top 5 ranked wash solvents are shown in Table 3.

**Table 3:** Ranked List of Wash Solvent Candidates Generated Removing the Solvents Showing a Warning Flag Related to Boiling and Melting Point Constraints and Solubility Constraints

			COSMO-RS calculated solubility (g/100 g)		
crystallization solvent	rank	wash solvent	paracetamol	metacetamol	acetanilide
2-propanol	1	heptane	<0.005	<0.005	0.04
	2	isopropyl acetate	2.73	6.31	11.20
	3	2-pentanol	2.35	3.60	8.73
	4	*tert*-butyl acetate	2.56	6.11	9.20
	5	1-octanol	1.65	2.54	6.68
ethanol	1	heptane	<0.005	<0.005	0.04
	2	methylisopropyl ketone	6.89	14.91	17.89
	3	propionic acid	5.20	6.55	42.73
	4	2-methyl-1-propanol	7.33	11.54	21.19
	5	butyric acid	4.58	6.00	37.96
3-methyl-1-butanol	1	dodecane	<0.005	<0.005	0.03
	2	2-pentanol	2.35	3.60	8.73
	3	ethanethiol	0.04	0.08	10.98
	4	1-octanol	1.65	2.54	6.68
	5	dimethoxyethane	0.42	0.80	7.27

**Figure 3:**
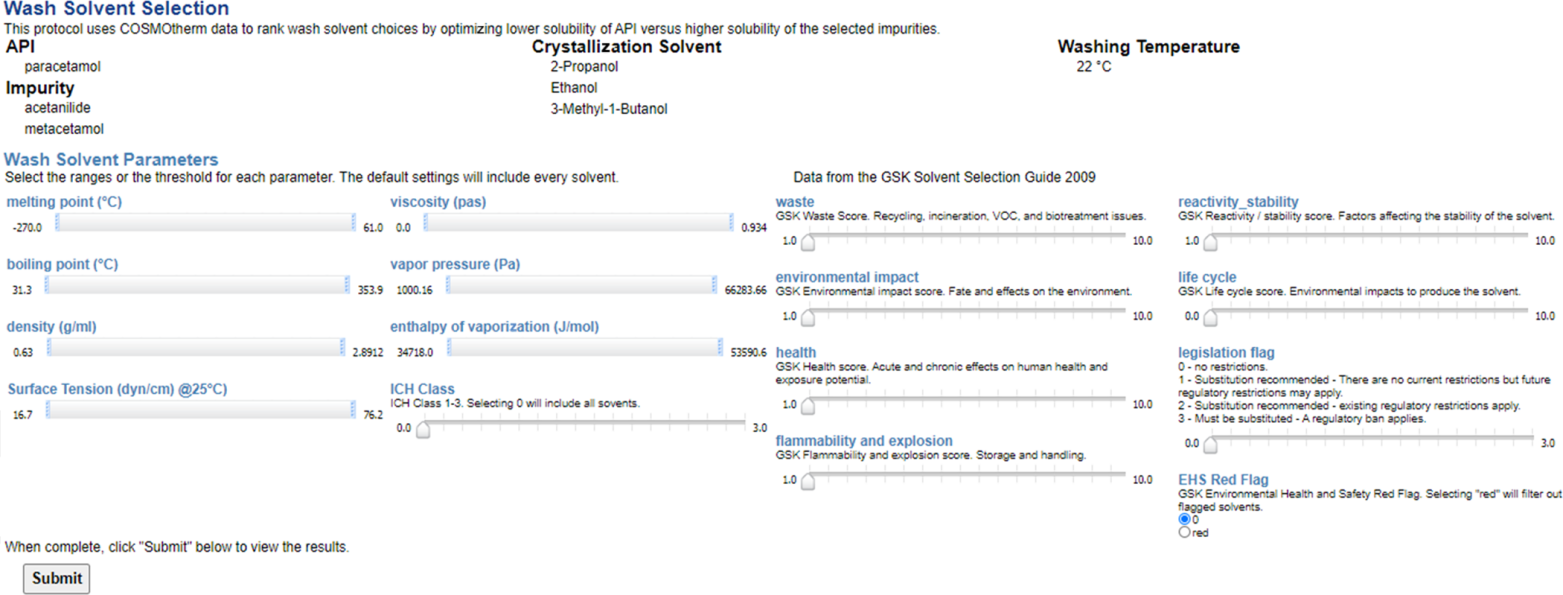
GUI interface to select the range of chemical, physical , health and safety, and environmental constraints to rank wash solvents.

To allow greater flexibility in the filtering and selection of wash solvents, a graphical user interface (GUI) was developed using Pipeline Pilot’s^55^ reporting tools. This interactive reporting tool allows the user to select appropriate API and impurity molecules from a list where the COSMO-RS solubility calculations have already been computed. If the molecule is not in this list, an option to automatically parameterize the molecule and run the calculations is available. Next, crystallization solvents can be selected and finally the washing temperature is chosen. If all data required are available within the database, the user is then presented with threshold selection screen (Figure 3). In the absence of data, protocols are automatically run, and the user is notified when the dataset is complete. The threshold selection screen allows the user to select acceptable ranges for a number of critical properties as well as categories contained within the GSK solvent selection guide. At any point, the selections can be submitted and a ranked lists of wash solvents is reported back to the user based on the multi-objective optimization discussed above. For additional detail on the GUI tool, see the Supporting Information.

The physical properties available for filtering include melting point, boiling point, density, surface tension, viscosity, vapor pressure, and enthalpy of vaporization. Values for these measurements have been gathered from the literature. In cases where the values are not available, these specific solvents are not subject to the specific filter and allowed to pass. An exception to this rule is the ICH filter. Selecting a specific threshold here will only pass solvents that have been assigned an ICH class of that or greater. Parameters derived within the GSK solvent selection guide 2009^56^ are also built in. These include

- Waste: covering recycling, incineration, VOC, and biotreatment issues;
- Environmental impact: covering the fate and effects of solvents on the environment;
- Health: covering acute and chronic effects on human health and the potential for exposure;
- Flammability and explosion: issues affecting storage and handling of solvents;
- Reactivity and stability: covering factors affecting the stability of the solvent;
- Life cycle: covering the environmental life cycle impacts from producing a solvent.

These are each scored from 1 to 10 with scores of 7–10 desirable and 1–3 flagged as major issues. Finally, legislation and environmental, health and safety-related legislation flags are included. This again allows the user to filter out potentially troublesome solvents.

The following table displays the result of increasing the number of filters applied within the wash solvent selection tool. Filter 1 applied no filtering of the data, and all 173 solvents were considered. Filter 2 removed immiscible solvents and solvents where the density was great than 1.3 times the density of the crystallization solvent. Solvents where the API solubility was great in the wash solvent were also removed. Filter 3 added the following physicochemical thresholds: melting point <0 °C, boiling point 60–130 °C, viscosity <0.09 Pa·s, and vapor pressure <10,000 Pa. Filter 4 includes all previous limits and sets ICH classification to 3 and all six GSK solvent selection criteria to >3.

It can be seen in Table 4 that the top 5 ranked wash solvents changes with increased parameter limitations. The wash solvent selection tool allows for quick and easy adjustment of all these parameters, thus enabling the researcher to select the desired solvent for validation in a logical fashion. Heptane is ranked first for 2-propanol and ethanol solvents, while dodecane is ranked first for 3-methyl-1-butanol using filter 3. However, increasing restrictions while applying limitations from the GSK solvent selection criteria (filter 4), heptane and dodecane are filtered out.

**Table 4:** The Top 5 Ranked Solvents for Each Crystallization Solvent while Applying Different Parameter Limitations

crystallization solvent	rank	filter 1	filter 2	filter 3	filter 4
2-propanol	1	perfluorohexane	heptane	heptane	isopropyl acetate
	2	*N*-methylformamide	trifluoroethanol	isopropyl acetate	4-fluorotoluene
	3	methanoic acid	isopropyl acetate	2-pentanol	2-pentanol
	4	methanol	dichloromethane	butyl acetate	butyl acetate
	5	acetone	2-pentanol	*tert*-butyl acetate	*tert*-butyl acetate
ethanol	1	perfluorohexane	heptane	heptane	4-fluorotoluene
	2	*N*-methylformamide	propionic acid	methyl isopropyl ketone	methyl isopropyl ketone
	3	methanoic acid	methyl isopropyl ketone	2-pentanol	2-pentanone
	4	methanol	1,3-dioxane	butyl acetate	2-pentanol
	5	acetone	dichloromethane	2-pentanone	2-butanol
3-methyl-1-butanol	1	perfluorohexane	dodecane	heptane	2-pentanol
	2	*N*-methylformamide	dichloromethane	2-pentanol	4-fluorotoluene
	3	methanoic acid	2-pentanol	butyl acetate	butyl acetate
	4	methanol	dimethyl carbonate	dimethoxyethane	diethyl carbonate
	5	acetone	thioacetic acid	diethyl carbonate	2,2-dimethoxypropane

Increasing the filters used for the solvent selection reduces the risk of unwanted effects during isolation (e.g., agglomeration, lumping, fine or impurity precipitation) and also improves the green metrics of the process. The ultimate decision of selecting a specific set of filters to identify the optimal solvent that suits all the relevant selection criteria is done by the practitioners, giving the practitioners the freedom of explore different alternative solvents. For example, an incoming material from a complex multi-stage synthesis may already have a large embedded environmental cost, meaning that the preservation of the yield is more important than minimizing the solvent consumption during the purification step.

### 3.2 Solvent Selection Tool Validation

As reported by Ottoboni *et al.*,^31^ a d-optimal DoE was performed to test which crystallization-wash solvent combination was able to maximize impurity removal during isolation while minimizing particle agglomeration. A series of crystallization (ethanol, isopropanol, and 3-methylbutan-1-ol) and wash solvents (*n*-heptane, isopropyl acetate, and *n*-dodecane) were used for the process. To mimic an industrial isolation process, two paracetamol related impurities, acetanilide and metacetamol, were dissolved in the mother liquor. Filter cake properties were determined using the on-board machine vision system in the CFD25 to halt filtration at dryland and to record filtration rate data. The filter cake and filtrate were both analyzed using HPLC to quantify the degree of purification achieved. The mechanical properties of the isolated product were evaluated; the extent of agglomeration, the agglomerate particle size distribution, and the agglomerate mechanical strength were all measured. Proton nuclear magnetic spectroscopy (^1^H NMR) was used to determine the residual solvent in the dried filter cake.

Twenty-one experiments with different combinations of crystallization and wash solvent were tested, and the best isolation strategy was achieved by washing the paracetamol cake crystallized from ethanol, with an ethanol–*n*-heptane mixture in wash 1 and then with pure *n*-heptane in wash 2 using a total of four void volumes of wash solvent (1 in washes 1 and 3 in wash 2). Both 2-propanol and ethanol were shown to be appropriate crystallization solvents. If 2-propanol is selected as the crystallization solvent, again the best wash solvent to use to minimize impurity retention and minimize particle agglomeration is *n*-heptane.

If 3-methyl-1butanol is selected as the crystallization solvent, *n*-heptane or *n*-dodecane can be considered as equivalently good candidates as the wash solvent, even although *n*-dodecane has a higher boiling temperature than *n*-heptane and so is more challenging to remove during drying.

### 3.3 Isolation Process Prediction

To extend the capability of this workflow to select optimal combination of wash solvent for a selected crystallization process (fixed crystallization solvent and a known content of impurities dissolved in the mother liquor), two modeling strategies were proposed here to predict the output of the selected crystallization-wash solvent isolation. Three different crystallization-wash solvent combinations were selected to test the two simulation tools used to model the composition of filtrate removed during washing (Table 5). These cases were selected as the best washing strategies predicted from the predictive tool without EHS constraints (Table 5), as reported in Section 3.1.

**Table 5:** Selected Crystallization-Wash Solvent Combination for the Solvent Selection Tool Validation

validation, experiment code from ref^34^	new experiment code	crystallization solvent	wash solvent
N1	Exp1	ethanol	*n*-heptane
N3	Exp2	2-propanol	*n*-heptane
N12	Exp3	3-methyl-1-butanol	*n*-dodecane

In the first step, the two different models were validated using three different experiments conducted by Ottoboni *et al.*,^31^ specifically Exp1 (ethanol and *n*-heptane case), Exp2 (2-propanol and *n*-heptane case), and Exp3 (3-methyl-1-butanol and *n*-dodecane case). Filtration and washing process conditions used are reported in Table 6. In the Supporting Information, the initial suspension and mother liquor mass fraction, the solvents properties used, and the particle size distribution of the two paracetamol grades used are reported.

**Table 6:** Experimental Parameters Used for Experiments N1, N3, and N12 of the DoE Experiment Conducted by Ottoboni *et al*.^31^

operative parameter	Exp1	Exp2	Exp3
volume slurry (mL)	120	140	120
solid mass (g)	14.202	16.506	24.3
crystallization solvent mass (g)	94.68	110.04	97.2
paracetamol solute mass (paracetamol dissolved in the mother liquor) (g)	13.681	8.932	4.921
dissolved acetanilide impurity solute mass (g)	0.499	0.455	0.522
dissolved metacetamol impurity solute mass (g)	0.558	0.509	0.584
solid load (%, w/w)	15	15	25
paracetamol particle grade	micronized	powder	powder
particle mean diameter D50 (μm)	13.85	77.36	77.36
cake porosity (%)	0.46	0.44	0.44
filtration and washing driving force (mbar)	200	200	200
number of washes (-)	2	2	2
equivalent cake void volume of wash solvent per each wash (-)	4	2	2
cake resistance^a^ (m/kg)	6.3 × 10^+08^	5.9 × 10^+07^	5.63 × 10^+08^

a Determined during the AWL CFD25 optimization DoE experiments. See Ottoboni *et al.*(31) and the Supporting Information.

The solubility of paracetamol, acetanilide, and metacetamol in the crystallization and wash solvent at isolation temperature (25 °C) was simulated with COSMO*therm* (see the Supporting Information).

The HPLC data of the filtrate removed after wash 1 and the filtrate removed after wash 2 are reported in Table 7.

**Table 7:** Mass of the Filtrate Removed in Experiments Exp1, Exp2, and Exp3 Reported in Ottoboni *et al*.^31^ after Wash 1 and Wash 2 and Mass of the Dissolved Species Contained in These Two Filtrates Phases^c^

	Exp1	Exp2	Exp3
mass of filtrate removed after W1 (mL)	10.79^c^	7.2765^d^	7.3^e^
concentration of paracetamol in filtrate after W1 (g/g filtrate)	0.2090	0.1087	0.0694
concentration of acetanilide in filtrate after W1 (g/g filtrate)	0.0080	0.0048	0.0044
concentration of metacetamol in filtrate after W1 (g/g filtrate)	0.0073	0.0037	0.0033
mass of filtrate removed after W2 (g)	31.306^f^	6.9964^g^	26.656^h^
concentration of paracetamol in filtrate after W2 (g/g filtrate)	0.0529	0.0911	0.0191
concentration of acetanilide in filtrate after W2 (g/g filtrate)	0.0018	0.0032	0.0025
concentration of metacetamol in filtrate after W2 (g/g filtrate)	0.0016	0.0026	0.0019

a Mass is calculated assuming density of the pure crystallization solvent.

b Mass is calculated using the density of the pure wash solvent.

c HPLC raw data are reported in the Supporting Information with the dilution calibration curves.

In Table 8, the percentage of impurities removed at the end of the washing for each experiment was calculated as the coefficient of variation of feed stream impurity mass and the residual mass of impurity at the end of wash 2.

**Table 8:** Percentage (%) of the Impurities Removed after the Final Washing Stage for Experiments Exp1, Exp2, and Exp3

	Exp1	Exp2	Exp3
acetanilide	99.64	99.86	99.04
metacetamol	99.72	99.89	99.35

The HPLC results reported in Table 8 show that the different samples analyzed are not completely free from impurities after the washing process. In particular, Exp2 (2-propanol as the crystallization solvent and *n*-heptane as the wash solvent) shows around 0.14% of residual acetanilide in the isolated cake and 0.11% of residual metacetamol after a washing process done with two washes, each one with an equivalent cake volume of 0.88, where the first wash is a mixture of 50–50% of 2-propanol and *n*-heptane. The worst case is for sample Exp3 (3-methyl-1-butanol as the crystallization solvent and *n*-dodecane as the wash solvent) where the residual acetanilide content in the cake is around 0.96% and the residual content of metacetamol is 0.65%. The washing strategy selected for sample Exp3, described in Table 6, demonstrates incomplete impurity removal for a washing process conducted with two washes, each of an equivalent cake volume of 0.88 (in total 1.76 equivalent cake volumes of wash solvent), where the first 0.88 cake volume fraction was a mixture of 20–80% of 3-methyl-1-butanol and *n*-dodecane, and the second wash was pure *n*-dodecane. As described by Ottoboni *et al.*,^31^ it was anticipated that experiment Exp1 would perform better than the other two cases since the washing strategy used was the most highly ranked. However, experiment Exp1, which employed micronized paracetamol, shows an intermediate amount of impurities removed with respect to the other two experiments, which used the powder grade of paracetamol. As reported, finer particles tended to migrate toward the filter medium reducing the void volume and increasing the tortuosity of the cake adjacent to the filter medium, slowing washing, potentially causing a much higher risk of impure mother liquor entrapment into the cake pores.^51^ This is believed to be the reason why the residual impurity content after washing is not the lowest, showing the residual acetanilide content equals to 0.36% and 0.28% for metacetamol.

#### 3.3.1 Model A Validation

To validate model A, the input stream composition and cake characteristics including porosity were matched with the input stream composition and the cake characteristics of the experimental samples Exp1, Exp2, and Exp3. The solubility of the paracetamol and the selected impurities in pure and mixed solvents was predicted using COSMO*therm.*^41,42^ The output of the simulation included the stream composition of the filtrates collected following the two different washing stages were simulated and compared with the HPLC results obtained by analysis of filtrate samples from experiments Exp1, Exp2, and Exp3 (section 3.3). The concentration of the different species determined by HPLC of the filtrate samples were converted from μg/mL to g/mL and to g/g of the total mass of the filtrate. To convert mL to g, it was assumed that the density of filtrate collected after wash 1 was equal to the density of the pure crystallization solvent and the density of filtrate collected after wash 2 corresponded to the density of the pure wash solvent. In Tables 9 and 10, the simulated impurities and dissolved paracetamol concentration reported as g/g of the total filtrate mass, were compared with the HPLC results. The filtration and washing yield and the total impurities removal achieved during washing (washes 1 and 2), obtained during the simulation, are compared with the experimental results to validate the goodness of fit of the model.

**Table 9:** Simulated mass fraction of paracetamol, acetanilide, and metacetamol of the input stream and filtrate collected after filtration and washing of Exp1. Simulated filtration and washing yield of Exp1, and simulated purity of Exp1. Simulation is done with model A

	simulated Exp1
Input stream	
paracetamol concentration solid and dissolved phase (g/g)	0.2599
acetanilide concentration (g/g)	0.0042
metacetamol concentration (g/g)	0.0052
Filtration	
paracetamol concentration removed (g/g)	0.1692
acetanilide concentration removed (g/g)	0.0034
metacetamol concentration removed (g/g)	0.0043
filtration yield (%)	46.04
Washing	
paracetamol concentration removed at 0.46 ECV (g/g)	0.0681
acetanilide concentration removed at 0.46 ECV (g/g)	0.0238
metacetamol concentration removed at 0.46 ECV (g/g)	0.0298
paracetamol concentration removed at 0.92 ECV (g/g)	0.0866
acetanilide concentration removed at 0.92 ECV (g/g)	0.0000
metacetamol concentration removed at 0.92 ECV (g/g)	0.0000
paracetamol concentration removed at 3.68 ECV (g/g)	0.1705
washing yield at 3.68 ECV (%)	36.86
removed acetanilide at 3.68 ECV (%)	100
removed metacetamol at 3.68 ECV (%)	100

**Table 10:** Simulated mass fraction of paracetamol, acetanilide, and metacetamol of the input stream and filtrate collected after filtration and washing of Exp2 and Exp3. Simulated filtration and washing yield of Exp2 and Exp3, and simulated purity of Exp2 and Exp3. Simulation is done with model A

	simulated Exp2	simulated Exp3
Input stream		
paracetamol concentration solid and dissolved phase (g/g)	0.1865	0.2292
acetanilide concentration (g/g)	0.0030	0.0037
metacetamol concentration (g/g)	0.0037	0.0046
Filtration		
paracetamol concentration removed (g/g)	0.0746	0.0478
acetanilide concentration removed (g/g)	0.0027	0.0017
metacetamol concentration removed (g/g)	0.0034	0.0021
filtration yield (%)	67.30	85.14
Washing		
paracetamol concentration removed at 0.44 ECV (g/g)	0.0812	0.0572
acetanilide concentration removed at 0.44 ECV (g/g)	0.0119	0.0000
metacetamol concentration removed at 0.44 ECV (g/g)	0.0149	0.0000
paracetamol concentration removed at 0.88 ECV (g/g)	0.0663	0.0482
acetanilide concentration removed at 0.88 ECV (g/g)	0.0000	0.0000
metacetamol concentration removed at 0.88 ECV (g/g)	0.0000	0.0000
paracetamol concentration removed at 1.76 ECV (g/g)	0.0383	0.0312
washing yield at 1.76 ECV (%)	60.90	77.87
removed acetanilide at 1.76 ECV (%)	100	100
removed metacetamol at 1.76 ECV (%)	100	100

From the simulations, the complete removal of the impurities was predicted in the case where the equivalent cake volumes of wash solvent used exceeded 0.88 for Exp2 and Exp3, while for Exp1, it was found through experimentation by design of experiments in which, to get a pure cake, it required an equivalent cake volume higher in excess of 0.92.

The predicted results for experiments Exp1, Exp2, and Exp3, respectively, as seen in Tables 9 and 10 show complete cake purification with all impurities removed well before the equivalent cake volumes measured experimentally and evaluated by HPLC as reported in section 3.4.

As reported in Section 2.2.2.2, model A comprises a series of assumptions that make it a simplistic tool capable of simulating an ideal displacement washing process, where complete mixing of the mother liquor and wash solvent is achieved only in the void volume of the cake. The wash solvent retained in the filter but above the cake, prior to being drawn into the cake is considered not mixed with the mother liquor in the cake. This level of approximations makes this model a low fidelity tool for simulating a real washing process since only pure displacement washing is considered without addressing diffusion. In a pure displacement, washing the wash solvent front penetrating the cake is assumed to be a plug flow flat-front, while in a real washing process, the wash front is known to have a typical finger front profile.^51^ Overall, model A is not appropriate to simulate real washing processes due to the lack of diffusion, dilution, or other washing mechanisms, and the simulation neglects solvents back-mixing or fingering wash front effects. Without the simulation of diffusion, the predicted washing efficiency is much higher than in a real process, causing the simulation of much higher purity product to be obtained (100% purity versus a lower purity measured in Exp1, Exp2, and Exp3, as reported in Table 8) using a lower content of wash solvent, as also described by Murugesan *et al.*, Beckmann, and Tien.^27,28,51^

#### 3.3.2 Model B Validation

Model B provides a more sophisticated prediction of filtration and washing than model A. As reported in Section 2.2.2.2, this model provides information about the filtrate and cake stream composition. It also simulates other filtration outcomes including filtration time, cake resistance, flow rate, etc. As part of the validation of model B, the input stream composition, cake characteristics including porosity, particle properties such as mean particle size and sphericity, and filtration outcome (cake resistance and media resistance) were matched to the input stream composition and the cake characteristics of the samples Exp1, Exp2, and Exp3. The solubility of the different compounds in single solvents were predicted using COSMO*therm.*^41,42^ In Tables 11 and 12, the impurities and dissolved paracetamol concentration is reported as g/g of the total filtrate mass for the simulation and for the HPLC results is compared. The filtration and washing yield and the total impurities removal achieved during washing (washes 1 and 2), obtained during the simulation, are compared with the experimental results to validate the goodness of fit of the model.

**Table 11:** Simulated mass fraction of paracetamol, acetanilide, and metacetamol of the input stream and filtrate collected after filtration and washing of Exp1. Simulated filtration and washing yield of Exp1, and simulated purity of Exp1. Simulation is done with model B

	simulated Exp1	experiment Exp1
Input stream		
paracetamol concentration solid and dissolved phase (g/g)	0.1250	0.2256
acetanilide concentration (g/g)	0.0046	0.0040
metacetamol concentration (g/g)	0.0051	0.0045
Filtration		
paracetamol concentration removed (g/g)	0.1250	0.2256
acetanilide concentration removed (g/g)	0.0046	0.0040
metacetamol concentration removed (g/g)	0.0051	0.0045
filtration yield (%)	57.37	63.10
Washing		
paracetamol concentration removed at 1.84 ECV (g/g)	0.6500	0.1290
acetanilide concentration removed at 1.84 ECV (g/g)	0.0237	0.0037
metacetamol concentration removed at 1.84 ECV (g/g)	0.0265	0.0048
paracetamol concentration removed at 3.68 ECV (g/g)	0.3618	0.0246
acetanilide concentration removed at 3.68 ECV (g/g)	0.0132	0.0002
metacetamol concentration removed at 3.68 ECV (g/g)	0.0147	0.0003
washing yield (%)	55.80	54.46
removed acetanilide (%)	95.70	96.71
removed metacetamol (%)	96.33	98.15

**Table 12:** Simulated mass fraction of paracetamol, acetanilide, and metacetamol of the input stream and filtrate collected after filtration and washing of Exp2 and Exp3. Simulated filtration and washing yield of Exp2 and Exp3, and simulated purity of Exp2 and Exp3. Simulation is done with model B

	simulated Exp2	experiment Exp2	simulated Exp3	experiment Exp3
Input stream				
paracetamol concentration solid and dissolved phase (g/g)	0.0745	0.1864	0.0477	0.2291
acetanilide concentration (g/g)	0.0038	0.0033	0.0051	0.0041
metacetamol concentration (g/g)	0.0042	0.0037	0.0057	0.0046
Filtration				
paracetamol concentration removed (g/g)	0.0745	0.1864	0.0477	0.2291
acetanilide concentration removed (g/g)	0.0038	0.0033	0.0051	0.0041
metacetamol concentration removed (g/g)	0.0042	0.0037	0.0057	0.0046
filtration yield (%)	68.86	71.91	85.68	67.92
Washing				
paracetamol concentration removed at 0.88 ECV (g/g)	0.3129	0.0666	0.2206	0.0452
acetanilide concentration removed at 0.88 ECV (g/g)	0.0159	0.0023	0.0234	0.0022
metacetamol concentration removed at 0.88 ECV (g/g)	0.0178	0.0030	0.0262	0.0029
paracetamol concentration removed at 1.76 ECV (g/g)	0.2983	0.0423	0.2096	0.0106
acetanilide concentration removed at 1.76 ECV (g/g)	0.0152	0.0012	0.0223	0.0003
metacetamol concentration removed at 1.76 ECV (g/g)	0.0170	0.0015	0.0249	0.0004
washing yield (%)	67.90	67.49	83.91	62.41
removed acetanilide (%)	93.94	95.84	91.55	95.81
removed metacetamol (%)	91.11	95.52	87.42	95.78

In Tables 11 and 12, the predicted and experimental liquid phase compositions are compared. Tables 11 and 12 present the predicted and measured levels of impurity removed from the product during filtration and during the two different washing stages. Filtration and wash yield was predicted in addition to the final purity at the end of the washing process. As seen from Tables 11 and 12, the amount of paracetamol removed during filtration and washing stages is comparable with the amount measured experimentally, therefore showing good match between simulated and measured filtration and filtration yield. As reported in these tables, the experimental yield obtained after filtration would be considered lower than the values expected for a commercial manufacturing process. The reason of these low yields is due to the crystallization solvent and conditions selected (solid load and isolation temperature). In Table 13, the crystallization solvent selection based on the predicted solubilities with the criteria reported in stages 1–4 of the solvent selection workflow. The crystallization solvents used for the validation of the workflow were chosen because of their widespread use,^57−60^ rather than being the preferable solvents to maximize the yield of the purification process. In Table 13, 3-methyl-1-butanol was ranked 13th, while 2-propoanol was ranked 26th and ethanol 34th as the potential crystallization solvent for paracetamol. Future work can be done to identify the effect of the best ranked crystallization solvent on the purification process performance.

**Table 13:** Top 10 Ranked Crystallization Solvents Suggested by the Solvent Selection Tool and the Rank Order of the Classical Crystallization Solvents Used to Crystallize Paracetamol^a^

rank	solvent	COSMO*therm* solubility at 22 °C (g/g)	return (g)	yield (%)
1	acetyl acetate	0.0060	36.13	98.36
2	3-pentanone	0.0100	35.56	97.27
3	butyl acetate	0.0104	34.48	97.06
4	water	0.0088	17.57	95.24
5	2-pentanol	0.0235	44.03	94.93
6	dimethyl carbonate	0.0134	23.41	94.59
7	methyl isobutyl ketone	0.0342	57.99	94.43
8	propionic acid	0.0520	70.41	93.13
9	formamide	0.1017	127.84	92.63
10	2-pentanone	0.0468	56.60	92.36
13	3-methyl-1-butanol	0.0482	51.65	91.47
26	2-propanol	0.0586	29.39	83.38
34	ethanol	0.1544	41.03	72.66

a Note that this ranking does not take account of potential chemical reactions with the product being crystallized or the toxicity and environmental desirability of the solvents modeled.

**Figure 4:**
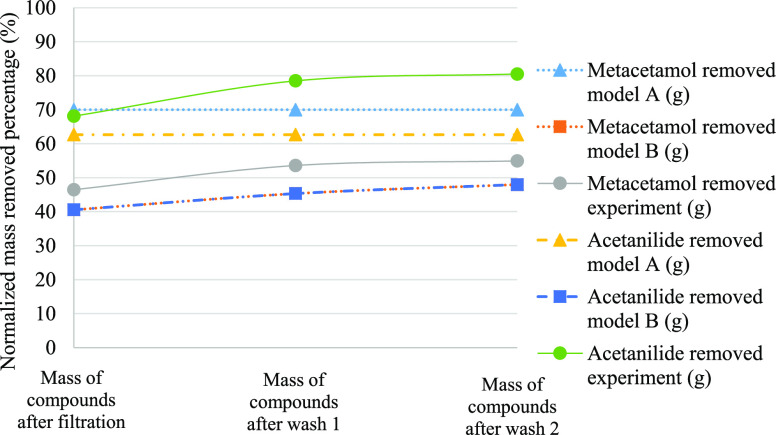
Comparison of simulated and experimental impurity rejection during filtration, wash 1, and wash 2 of Exp1.

**Figure 5:**
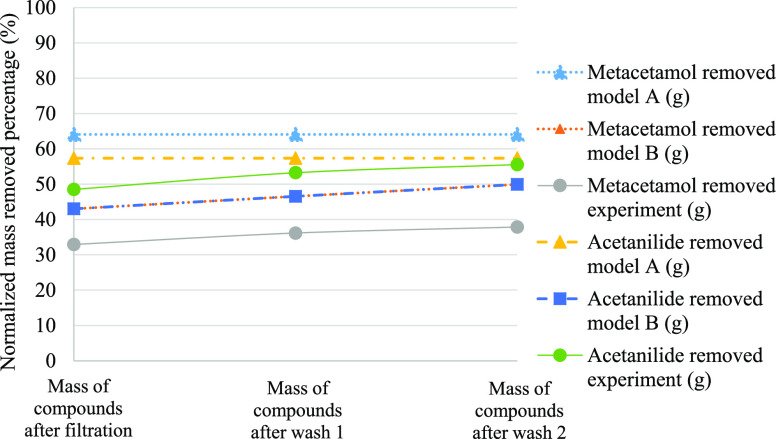
Comparison of simulated and experimental impurity rejection during filtration, wash 1, and wash 2 of Exp2.

**Figure 6:**
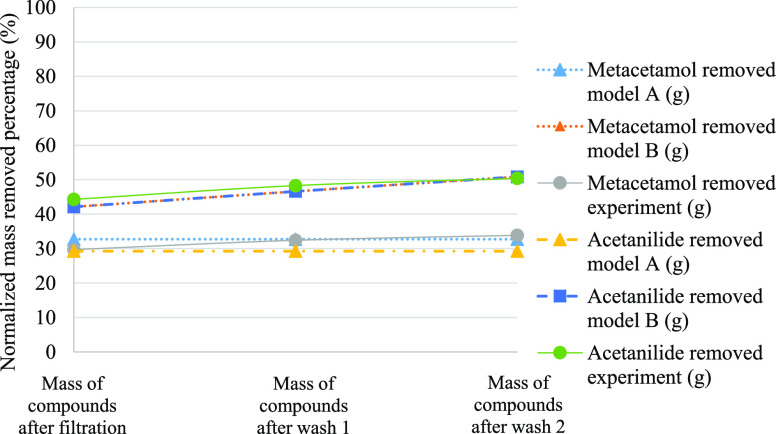
Comparison of simulated and experimental impurity rejection during filtration, wash 1, and wash 2 of Exp3.

The simulated amounts of impurities removed during filtration are comparable with the values measured for Exp1, Exp2, and Exp3, while the values of removed impurities during the first and second stage of washing are not comparable with the measured amount of impurities removed experimentally. The discrepancy observed for the impurity removal during washing can be attributed to the lack in the model of a solubility equation for acetanilide and metacetamol for the gradient mix solvent composition used to identify the variation of solubility across the washing stage. In our ongoing work, we are going to take account of the variation in solubility in binary solvents at varying wash solvent concentrations to simulate the variation of solvent composition in the cake from pure mother liquor to pure wash solvent. However, since in the literature no integrated filtration and washing models are reported so far, we consider the modeling approach B as an improvement of the existing modeling capability to simulate purification of APIs using a purely digital approach. In future work, we will increase the sophistication of the model by including the kinetics of dissolution. Overall, as seen from the value of purity achieved, the simulated amount of impurity removed during washing is lower but in the same order of magnitude than the amount of impurities measured for the different experiments. Since the discrepancy of simulated and measured values is less than an order of magnitude, they can be considered reasonably comparable, and model B is an appropriately sophisticated modeling tool to simulate washing performance in terms of impurity removal. To confirm that model B is better than model A to simulate filtration and washing performances, a comparison of impurity removal across model A, model B, and the measured experimental results is reported in Figures 4, 5, and 6. In these figures, it is clear that model B better predicts the purity across wash 1 and wash 2 of Exp1, Exp2, and Exp3.

#### 3.3.3 Model B: Simulation of Optimal Isolation

Building on the success of model B in predicting experimental outcomes of washing, it was decided to evaluate model B as a tool for optimizing the washing process. Ottoboni *et al.*(31) reported experimental optimization of washing of paracetamol powder and micronized paracetamol in the presence of the impurities acetanilide and metacetamol. This was achieved experimentally by washing the cake with at least 3 separate aliquots of wash solvent, each aliquot of at least 4 equivalent cake void volumes for the powder grade of paracetamol, where the first wash aliquot was a mixture of pure crystallization and wash solvent (to prevent the anti-solvent effect precipitating impurities), while the subsequent washes were pure wash solvent.

Four different cases were simulated to evaluate the solvent selection tool in combination with the isolation process modeling tool (model B). The overarching objective was to determine whether this approach could be used as a prediction first tool to facilitate isolation process design and to minimize waste generation during R&D for a new candidate molecule. Here, two of the cases employing 2-propanol as the crystallization solvent and n-heptane as the final wash solvent are presented. Two additional cases, where ethanol and *n*-heptane were selected as the crystallization solvent and wash solvent, are reported in the Supporting Information.

These simulations used, as initial input conditions, the suspension characteristics and the filtration performances measured in experiment Exp2.

Case 1 simulates filtration and washing of powder grade paracetamol where 3 washes were performed each using 1.76 equivalent cake volumes of wash solvent (equivalents to 2 cake void volumes). Wash 1 comprised a solvent mixture of 50-50% of 2-propanol and *n*-heptane to prevent the anti-solvent effect.^31,53^ In case 2, a micronized cake was used to simulate a filtration and washing experiment where 3 washes were conducted each comprising 1.84 equivalent cake volume of wash solvent (equivalents to 3 cake void volume). The same solvent compositions were used as for case 1.

In Tables 14 and 15, the simulated values of impurity and dissolved paracetamol concentration are reported as g/g of the total filtrate mass for the filtrate samples removed during filtration, washes 1, 2, and 3, as well as the final product purity achieved. These recommended washing strategies are based purely on simulation using model B justified on the basis of the goodness of fit of the simulation shown in Section 3.3.2. as a validation.

**Table 14:** Simulated Concentration of Paracetamol, Acetanilide, and Metacetamol Removed during Filtration, Washes 1, 2, and 3 (Collected in the Filtrate Phase) of Isolation Optimal Strategy Case 1, Filtration and Washing Yield (%), and Purity Achieved Corresponds to 98.73%

simulated case 1	concentration in filtrate collected after filtration (g/g filtrate)	concentration in filtrate collected after W1 (g/g filtrate)	concentration in filtrate collected after W2 (g/g filtrate)	concentration in filtrate collected after W3 (g/g filtrate)
paracetamol	0.0745	0.3793	0.2192	0.1247
acetanilide	0.0038	0.0193	0.0112	0.0064
metacetamol	0.0042	0.0216	0.0125	0.0071
yield (%)	68.86	66.31	63.87	61.55

**Table 15:** Simulated Concentration of Paracetamol, Acetanilide, and Metacetamol Removed during Filtration, Washes 1, 2, and 3 (Collected in the Filtrate Phase) of Isolation Optimal Strategy Case 2, Filtration and Washing Yield (%), and Purity Achieved Corresponds to 98.78%

simulated case 2	concentration in filtrate collected after filtration (g/g filtrate)	concentration in filtrate collected after W1 (g/g filtrate)	concentration in filtrate collected after W2 (g/g filtrate)	concentration in filtrate collected after W3 (g/g filtrate)
paracetamol	0.0745	0.3963	0.2190	0.1191
acetanilide	0.0038	0.0202	0.0112	0.0061
metacetamol	0.0042	0.0226	0.0125	0.0068
yield (%)	68.86	65.65	63.30	61.61

## 4 Conclusions

The wash solvent selection methodology seeks to preserve the desirable particle attributes generated during crystallization by taking account of the risk of precipitation and particle dissolution during washing. The workflow also prioritizes solvents that are favorable for drying. The workflow procedure is designed to allow practitioners to digitally design a purification strategy for NCEs minimizing the risk of changes to particle properties during isolation while maximizing the purity of the final isolated product using benign solvents.

The digital solvent selection and isolation performance prediction tool achieves this by narrowing the wide range of possible solvent and process choices down to a limited list of well selected options, which can then be validated experimentally. This increases R&D productivity and reduces the amount of waste generated during process development. Six of the nine stages in the workflow address the selection of crystallization and wash solvent using predicted solubility and other relevant solvent properties (e.g., safety, density, viscosity, and thermodynamic properties). The remaining stages are related to the isolation performance prediction.

The workflow has been exemplified using COSMO*therm* as the solvent prediction tool, but any other solubility prediction software could be used. A key element of the workflow is the digital tool used to rank the isolation solvents into a list of good candidates to evaluate experimentally.

Experimental data has been from the same research group, Ottoboni *et al.*,^31^ and was used to demonstrate the approach to validation. Another important element of the methodology is the layering of models with increasing sophistication, and this is exemplified with models A and B. Model A predicted complete removal of impurities from the filter cake well before the required equivalent cake volumes of wash measured experimentally by HPLC results. The assumptions used in model A were shown to be too simplistic to be useful in simulating the process. Model B showed good agreement with the experimental data, successfully predicting the extent of impurity removal achieved, during each washing step, the results being comparable with the experimental data.

Overall, the proposed solvent selection workflow has been shown to be a versatile prediction tool for solvent selection supporting digital process design. It is capable of transferring material property information generated using a combination of published material properties and predictions between simulated unit operations with the goal of selecting the ideal purification strategy based on testing then the likely performance of the isolation process selected via simulation. This solvent selection workflow is therefore a versatile “prediction first” tool to use for both NCEs and existing compounds to digitally design purification strategies with the aim of reducing the experimental resource consumed and waste material produced during purification process development.

## Supporting Information Available

(Table S1) Solvent used for the solvent selection and their chemical and physical properties;^61^ (Table S2) COSMO-RS predicted solubility of paracetamol, acetanilide, and metacetamol at isolation temperature in the different solvents used for the solvent selection; (Table S3) slurry (suspension) mass fraction of the experiments N1, N3, and N12 reported by Ottoboni *et al*.^31^ used for the filtration and washing models validation stage; (Table S4) mother liquor mass fraction of the experiments N1, N3, and N12 reported by Ottoboni *et al*.^31^ used for the filtration and washing models validation stage; (Table S5) solvent properties used in model B to simulate filtration and washing process;^31^ (Figure S1) cumulative distribution of the particle size analysis of micronized paracetamol; (Figure S2) cumulative distribution of the particle size analysis of powder paracetamol; (Table S6) mean volumetric particle size and standard deviation of the particles size distribution of the micronized and powder paracetamol used for the filtration and washing model B used for the prediction of isolation performances; (Table S7) paracetamol, acetanilide, and metacetamol COSMOtherm solubility prediction in ethanol, 2-propanol, 3-methyl-1-butanol, *n*-heptane, and *n*-dodecane at isolation temperature (25 °C); (Table S8) paracetamol, acetanilide, and metacetamol solubility in ethanol, 2-propanol, 3-methyl-1-butanol, *n*-heptane, and *n*-dodecane at isolation temperature (25 °C) experimentally measured with the gravimetric method; (Figure S3) COSMOtherm binary plot solubility of paracetamol (API), acetanilide, and metacetamol in a gradient crystallization solvent (2-propanol) and wash solvent mixture (*n*-heptane); (Figure S4) COSMOtherm binary plot solubility of paracetamol (API), acetanilide, and metacetamol in a gradient crystallization solvent (ethanol) and wash solvent mixture (*n*-heptane); (Figure S5) COSMOtherm binary plot solubility of paracetamol (API), acetanilide, and metacetamol in a gradient crystallization solvent (3-methyl-1-butanol) and wash solvent mixture (*n*-dodecane); (Figure S6) HPLC calibration curve of paracetamol; (Figure S7) HPLC calibration curve of metacetamol and acetanilide; (Table S9) HPLC peak areas and concentration of paracetamol, acetanilide, and metacetamol dissolved in the filtrate sample collected after wash 1 and wash 2 of experiments N1, N3, and N12 of the DoE reported by Ottoboni *et al*.;^31^ (Table S10) predicted filtration and washing parameter obtained from model B; (Table S11) simulated concentration of paracetamol, acetanilide, and metacetamol removed during washes 1, 2, and 3 (collected in the filtrate phase) of isolation optimal strategy case 3; and (Table S12) simulated concentration of paracetamol, acetanilide, and metacetamol removed during washes 1, 2, and 3 (collected in the filtrate phase) of isolation optimal strategy case 4 (PDF)

## Abbreviations

API: active pharmaceutical ingredient
CQA: critical quality control
PSD: particle size distribution
ICH: International Harmonisation Congress
LOD: loss on drying
DoE: design of experiment
HPLC: high-performance liquid chromatography
RI: refractive index detectors
GSK: Glaxo Smith Kline
GUI: graphical user interface
^1^H NMR: proton nuclear magnetic resonance
NCE: new compound entity
R&D: research and development

## Footnotes

a With the term filtration stopped to dryland, it is considered the halting of the filtration that ensures that the cake is fully saturated with the mother liquor. Halting filtration to dryland makes cracking unlikely but retains the impure mother liquor in all the interparticulate pores. Alternatively, filtration to breakthrough occurs when the cake is deliquored sufficiently for air or nitrogen from above the cake to form bubbles on the low pressure side of the medium supporting the cake. In this case, more of the impure mother liquor is removed, but the cake is very likely to have cracks running all the way through from top to bottom, making subsequent washing much less effective.
