# Supplementary material for: A Novel Integrated Workflow for Isolation Solvent Selection Using Prediction and Modeling
Source: Org Process Res Dev. 2021 May 5;25(5):1143–59. doi: 10.1021/acs.oprd.0c00532 (PMC8289338; doi:10.1021/acs.oprd.0c00532)
Supplement: Supplementary file 1 — op0c00532_si_001.pdf [file op0c00532_si_001.pdf]

Supplementary information: A novel integrated workflow for isolation solvent selection using prediction and modelling

Sara Ottoboni[1,2], Bruce Wareham[3], Antony Vassileiou[3], Murray Robertson[3], Cameron J. Brown[3], Blair Johnston[3,4,5] Chris J. Price[1,2]*

[1]EPSRC Centre for Innovative Manufacturing in Continuous Manufacturing and Crystallisation, University of Strathclyde, 99 George Street, Glasgow, G1 1RD, UK

[2]Department of Chemical and Process Engineering, University of Strathclyde, 75 Montrose Street, Glasgow, G1 1XL, UK

[3]Strathclyde Institute of Pharmacy & Biomedical Science (SIPBS), University of Strathclyde, 99 George Street, Glasgow, G1 1RD, UK

[4]Strathclyde Institute of Pharmacy & Biomedical Science (SIPBS), University of Strathclyde, 161 Cathedral Street, Glasgow, G4 0RE, UK

[5] National Physical Laboratory, Hampton Road, Teddington, Middlesex, TW11 0LW, UK

Table S1 Solvent used for the solvent selection and their chemical and physical properties[61].

Solvent Name	Melting Point (°C)	Boiling Point (°C)	Density (g/ml)	Surface Tension (dynes/cm 25 °C)	Viscosity (Pas)	Enthalpy of Vaporisation (J/mol)	Vapor Pressure (Pa)	Waste	Environmental Impact	Health	Flammability and Explosion	Reactivity Stability	Life Cycle Score	Legislation Flag	EHS Red Flag	ICH Class
1,1,1-Trichloroethane	-33	74	1.3376	25	7.90E-04											1
1,1-Dichloroethene	-122	32	1.175													1
1,1-Diethoxypropane	-100	122.8	0.815													
1,2,3-Trimethoxypropane	-270	143.1	0.912													
1,2,4-Trichlorobenzene	17	214	1.454					7	4	4	9	10	8	3	red	
1,2-Dichlorobenzene	-17	180	1.306	35.7	1.32E-03			7	4	6	10	9	8	0	red	
1,2-Dichloroethane	-36	84	0.87	32.6	7.80E-04			4	4	2	6	10	7	1	red	1
1,2-Propanediol	-60	188	1.036	45.6	4.04E-02			6	6	10	10	10	3	0	0	
1,3,5-Trimethylbenzene	-45	165	0.864					8	3	7	6	10	7	0	0	
1,3-dimethyltetrahydropyrimidin-2(1H)-one	-23	247	1.06					7	7	4	9	7	3	3	0	
1,3-Dioxane	-42	105	1.032													
1,3-Propanediol	-27	214	1.06					6	6	9	10	10	3	0	0	
1,4-Butanediol	20	235	1.017					6	6	8	10	10	4	0	0	
1,4-Dimethylbenzene	-13	138	0.86104	27.9	6.00E-04			7	2	6	5	10	7	0	0	

Solvent Name	Melting Point (°C)	Boiling Point (°C)	Density (g/ml)	Surface Tension (dynes/cm 25 °C)	Viscosity (Pas)	Enthalpy of Vaporisation (J/mol)	Vapor Pressure (Pa)	Waste	Environmental Impact	Health	Flammability and Explosion	Reactivity Stability	Life Cycle Score	Legislation Flag	EHS Red Flag	ICH Class
1,4-Dioxane	12	102	1.03	32.9	1.18 E-03			3	4	4	4	5	6	2	0	2
1,5-Pentanediol	-18	242	0.994													
1-Bromo-2-Chloroethane	-18	106.5	1.723													
1-Bromobutane	-112.5	99	1.276													
1-Butanol	-89	118	0.81	25	2.54 E-03		1000.16	5	7	5	8	9	5	0	0	3
1-Chlorobutane	-123.1	78	0.886													
1-Hexanol	-52	157	0.82													
1-Methylnaphthalene	-22	240	1.0202													
1-Octanol	-16	195	0.8262													
1-Pentanol	-78	137	0.814													3
1-Propanol	-127	97	0.803	20.9	1.95 E-03	47438.5	2786.98	4	7	5	7	10	7	0	0	3
Perfluoro-2-butyltetrahydrofuran	-88	103	1.77					5	2	3	7	10	0	0	red	
2,2,4-Trimethylpentane	-107	99	0.692					6	4	8	3	10	7	0	0	
2,2-Dimethoxypropane	-47	83	0.847													
2-(2-Hydroxyethoxy)Ethanol	-10	246	1.118	55.1	3.02 E-02			6	8	7	9	9	8	0	0	
2-[2-(2-Hydroxyethoxy)Ethoxy]Ethanol	-7	285	1.124					6	8	6	10	9	7	0	0	
2-Amino-1-Butanol	-2	176	0.944													

Solvent Name	Melting Point (°C)	Boiling Point (°C)	Density (g/ml)	Surface Tension (dynes/cm 25 °C)	Viscosity (Pas)	Enthalpy of Vaporisation (J/mol)	Vapor Pressure (Pa)	Waste	Environmental Impact	Health	Flammability and Explosion	Reactivity Stability	Life Cycle Score	Legislation Flag	EHS Red Flag	ICH Class
2-Bromobutane	-112.7	91	1.255													
2-Butanol	-115	100	0.806	22.6	3.05 E-03	49143.3	2366.03	4	6	8	7	9	6	0	0	3
2-Butanone	-87	80	0.805	24	4.10 E-04	34718	12192.5	3	7	8	4	8	3	0	0	3
2-Butoxyethanol	-77	171	0.902	26.6												
2-Chloroacetic Acid	61	189	1.404					4	6	6	10	8	7	0	0	
2-Ethoxy-2-Methylpropane	-74	70	0.742					5	5	4	4	9	8	0	0	
2-Ethoxyethanol	-70	135	0.9253	28.8												2
2-Ethylhexanol	-76	185	0.8344	27.7	6.27 E-03			9	5	6	9	10	6	0	0	
2-Iodobutane	-104	119	1.598													
2-Methoxy-2-Methylbutane	-80	86	0.77					5	5	5	5	9	8	0	0	
2-Methoxy-2-Methylpropane	-109	55	0.7404					4	5	5	3	9	8	0	red	3
2-Methoxyethanol	-85	124	0.965	42.8				3	8	2	7	6	7	2	red	2
2-Methoxyethylether	-68	162	0.943					4	5	2	8	4	6	2	red	
2-Methyl-1-Propanol	-101.9	107.89	0.8018	22.6	3.35 E-03	52300	1516.32									3
2-Methylbutan-2-ol	-9	102														
2-Methylpentane	-153	60	0.653					5	4	7	2	10	7	0	0	
2-Methylpropan-2-ol	25	82	0.775					3	9	6	6	10	8	0	0	
2-Methyltetrahydrofuran	-137	78	0.86					4	5	4	3	6	4	0	0	

Solvent Name	Melting Point (°C)	Boiling Point (°C)	Density (g/ml)	Surface Tension (dynes/cm 25 °C)	Viscosity (Pas)	Enthalpy of Vaporisation (J/mol)	Vapor Pressure (Pa)	Waste	Environmental Impact	Health	Flammability and Explosion	Reactivity Stability	Life Cycle Score	Legislation Flag	EHS Red Flag	ICH Class
2-Octanol	-38	179	0.8193													
2-Pentanol	-50	119	0.812		3.45 E-03	53590.6		6	6	6	8	8	6	0	0	
2-Pentanone	-78	102	0.809			38400	4720	5	6	6	7	10	4	0	0	
2-Phenylethanol	-27	219	1.0202													
2-Propanethiol	-130	57	0.82													
2-Propanol	-88	82	0.785	23.3	2.04 E-03			3	9	8	6	8	4	0	0	3
3-Fluorotoluene	-87	114	0.991													
3-Methyl Thiophene	-69	115	1.016													
3-Methyl-1-Butanol	-117	131	0.809					6	6	7	9	10	6	0	0	3
3-Pentanone	-42	102	0.813		4.48 E-04	37220.02	4723	5	6	8	7	6	4	0	0	
4-Fluorotoluene	-56	116	1													
4-Methyl-1-Pentanol	-69.95	151.9	0.821													
5-Methyl furfural	-11	187	1.107													
Acetic Acid	17	118	1.05	27	1.06 E-03			4	8	6	8	7	8	0	0	3
Acetone	-95	56	0.791	23	3.10 E-04			3	9	8	4	9	7	0	0	3
Acetonitrile	-45	82	0.982	28.7	3.70 E-04			2	6	6	6	10	3	0	0	2
Acetyl Acetate	-73	140	1.08					5	8	4	8	6	6	0	0	
Aniline	-6.3	184.13	1.022	42.4	3.85 E-03											
Anisole	-38	154	0.995					6	6	7	7	6	5	0	0	3
Benzene	6	80	0.879	28.2	6.00 E-04			5	6	1	3	10	7	2	red	1
Benzylalcohol	-15	205	1.0419	36.8	5.47 E-03			6	6	7	10	7	6	0	0	

Solvent Name	Melting Point (°C)	Boiling Point (°C)	Density (g/ml)	Surface Tension (dynes/cm 25 °C)	Viscosity (Pas)	Enthalpy of Vaporisation (J/mol)	Vapor Pressure (Pa)	Waste	Environmental Impact	Health	Flammability and Explosion	Reactivity Stability	Life Cycle Score	Legislation Flag	EHS Red Flag	ICH Class
Bromobenzene	-30.8	156	1.491													
Bromoform	8	150	2.8912													
Butyl Acetate	-77	126	0.68	24.8	6.90 E-04			7	7	8	8	10	5	0	0	3
Butyl Lactate	-28	188	0.9744													
Butyric Acid	-5.1	163.75	0.9528													
Butyronitrile	-111.9	117.6														
Carbon Tetrachloride	-23	77	1.594	26.3	9.10 E-04			4	5	3	4	10	7	1	red	1
Chlorobenzene	-45	132	1.1058					6	6	4	8	10	8	0	0	2
Chloroform	-64	61	1.492	26.7	5.40 E-04			3	6	3	6	9	6	2	red	2
cis-Decalin	-43	196	0.897					7	3	7	6	7	7	0	0	
Cumene	-96	152	0.864					7	5	6	8	5	7	0	0	2
Cyclohexane	7	81	0.7781	24.7	8.90 E-04			5	5	7	2	10	7	0	0	2
Cyclohexanol	25	161	0.948	33.4	5.75 E-02			6	6	7	9	9	8	0	0	
Cyclohexanone	-32	155	0.947	34.4	2.02 E-03			6	8	6	8	9	6	0	0	
Cyclopentane	-93.9	49.2	0.751													
Cyclopentanone	-51	131	0.951					7	6	6	8	10	6	0	0	
Cyclopentyl Methyl Ether	-140	106	0.86				4573	6	4	4	5	8	4	0	0	
Dibutylether	-95	140	0.764					7	7	4	5	5	4	0	0	
Dichloromethane	-95	40	1.325	27.8	4.10 E-04			3	6	4	6	9	7	2	red	2
Diethylcarbonate	-43	126	0.975													

Solvent Name	Melting Point (°C)	Boiling Point (°C)	Density (g/ml)	Surface Tension (dynes/cm 25 °C)	Viscosity (Pas)	Enthalpy of Vaporisation (J/mol)	Vapor Pressure (Pa)	Waste	Environmental Impact	Health	Flammability and Explosion	Reactivity Stability	Life Cycle Score	Legislation Flag	EHS Red Flag	ICH Class
Diethylene Glycol Butyl Ether	-68	231	0.95					6	7	7	9	6	7	0	0	
Diethylether	-116	35	0.706	16.7	2.20 E-04			4	4	5	2	4	6	0	red	3
Diethylsulfide	-103.8	92	0.8362													
Dimethoxyethane	-58	85	0.867	20				4	5	2	4	4	7	2	red	
Dimethoxymethane	-105	42	0.867	18.8												
Dimethyl Carbonate	-1	91	1.069					4	8	7	6	10	8	0	0	
Dimethyl Disulfide	-85	110	1.0625													
Dimethyl Sulfide	-98	35	0.846													
Dimethylsulphoxide	19	189	1.1	42.9	1.99 E-03			5	5	7	9	2	6	0	0	3
Dodecane	-10	214	0.7495													
Ethanethiol	-148	35	0.839													
Ethanol	-114	78	0.789	22	1.07 E-03			3	8	8	6	9	9	0	0	3
Ethyl Acetate	-84	77	0.902	23.2	4.20 E-04			4	8	8	4	8	6	0	0	3
Ethyl Formate	-80	54	0.921					4	6	5	4	9	0	0	red	3
Ethyl Isovalerate	-99	131	0.864													
Ethyl Lactate	-23	154	1.0328					7	5	4	8	10	0	0	red	
Ethyl Phenyl Ether	-29	170	0.966					8	4	7	10	10	0	0	red	
Ethyl Propanoate	-74	99	0.884					5	7	4	6	6	0	0	red	
Ethylene Carbonate	36	248	1.321					6	7	5	10	9	0	0	red	

Solvent Name	Melting Point (°C)	Boiling Point (°C)	Density (g/ml)	Surface Tension (dynes/cm 25 °C)	Viscosity (Pas)	Enthalpy of Vaporisation (J/mol)	Vapor Pressure (Pa)	Waste	Environmental Impact	Health	Flammability and Explosion	Reactivity Stability	Life Cycle Score	Legislation Flag	EHS Red Flag	ICH Class
Ethylene Glycol	-13	197	1.113	48.4	1.61E-02			5	8	7	10	9	9	0	0	2
Fluorobenzene	-42	85	1.024					5	3	6	5	9	1	0	0	
Formamide	3	220	1.134	57	3.34E-03			4	7	2	10	8	8	2	0	2
Furan	-85.6	31.3	0.9731													
Furfural	-37	162	1.16													
Furfuryl Alcohol	-29	170	1.135	53.3												
Glycerol	18	290	1.261	76.2	0.934			6	7	8	10	9	8	0	0	
Glycerol Carbonate	-270	353.9	1.39													
Heptane	-91	98	0.68	19.8	3.90E-04			6	3	8	3	10	7	0	0	3
Hexane	-95	69	0.659					5	3	4	2	10	7	3	red	2
Iodobenzene	-29	188	1.808													
Iodomethane	-66.5	42.4	2.28													
Isoamyl Acetate	-78.5	142.5	0.876													
Isobutyl Acetate	-99	118	0.87	23	6.80E-04											3
Isopropyl Acetate	-73	89	0.872	22.3				5	7	7	6	9	7	0	0	3
Isopropyl butadecanoate	-17.5	248														
Isopropyl Ether	-86	68	0.725					4	3	8	1	1	9	0	red	
Limonene	-74.35	176	0.84													
Methanedithione	-111	46	1.266					4	6	2	1	6	8	3	red	
Methanoic Acid	8.4	100.8	1.22	37.7	1.61E-03											3
Methanol	-98	65	0.791	22.1	5.40E-04			4	9	5	5	10	9	0	0	2

Solvent Name	Melting Point (°C)	Boiling Point (°C)	Density (g/ml)	Surface Tension (dynes/cm 25 °C)	Viscosity (Pas)	Enthalpy of Vaporisation (J/mol)	Vapor Pressure (Pa)	Waste	Environmental Impact	Health	Flammability and Explosion	Reactivity Stability	Life Cycle Score	Legislation Flag	EHS Red Flag	ICH Class
Methyl Acetate	-98	57	0.9342	24.5	3.60 E-04			3	9	7	4	9	7	0	0	3
Methyl Isobutyl Ketone	-84	117	0.801	23.5	5.42 E-04	40576.6667	2619.78	6	6	6	7	8	2	0	0	3
Methyl Lactate	-66	144	1.093					5	9	4	8	9	5	0	0	
Methylbutyl Ketone	-55.5	127.6	0.812													2
Methylcyclohexane	-127	101	0.77					6	5	8	3	10	7	0	0	2
Methylcyclopentane	-142	72	0.749					6	4	5	2	9	7	0	0	
Methylisopropyl Ketone	-92	92	0.805													
N,N-Dimethylacetamide	-20	165	0.937					5	6	2	10	8	2	2	red	2
N,N-Dimethylaniline	3	194	0.956					7	5	4	8	8	3	2	red	
N,N-Dimethylformamide	-61	153	0.944	34.4	7.90 E-04			4	6	2	9	9	7	2	red	2
N-Methyl-2-Pyrrolidone	-24	202	1.028	44.6				5	6	3	9	8	4	2	red	2
N-Methylformamide	-4	200	1.011					4	6	2	10	10	7	2	red	
Nitrobenzene	5.7	210.9	1.2037													
Nitromethane	-29	101	1.127	36.3	6.30 E-04			3	8	4	7	2	2	2	red	2
Octyl Acetate	-39	210	0.868					9	5	5	8	10	6	0	0	
Pentane	-130	36	0.63		2.21 E-04		66283.66	5	6	8	2	10	7	0	red	3
Pentylacetate	-71	149	0.8756													

Solvent Name	Melting Point (°C)	Boiling Point (°C)	Density (g/ml)	Surface Tension (dynes/cm 25 °C)	Viscosity (Pas)	Enthalpy of Vaporisation (J/mol)	Vapor Pressure (Pa)	Waste	Environmental Impact	Health	Flammability and Explosion	Reactivity Stability	Life Cycle Score	Legislation Flag	EHS Red Flag	ICH Class
Perfluorocyclohexane	51	53	1.68					5	5	3	5	10	0	0	red	
Perfluorohexane	-86	57	1.669					4	4	3	5	10	0	3	red	
Perfluorotoluene	-66	104	1.668					5	3	4	5	10	0	0	red	
Phenoxybenzene	27	258	1.073					8	5	4	8	6	0	0	red	
Piperidine	-11.03	106.2	0.862													
Propanenitrile	-93	97	0.772					3	6	4	6	9	0	0	red	
Propionic Acid	-21	141	0.993					4	8	6	8	8	7	0	0	
Propyl Acetate	-92	102	0.888	23.9	5.66 E-04	39455	4603.62	5	7	8	6	10	4	0	0	3
Propylene Carbonate	-55	242	1.2047					6	7	5	8	9	0	0	red	
Pyridine	-42	115	0.978	36.7	8.80 E-04			3	4	4	7	9	2	2	red	2
Sulfolane	28	282	1.261					5	9	6	10	10	0	0	red	2
Tert-Butyl Acetate	-78	95	0.8665					6	9	8	6	10	8	0	0	
Tetrachloroethene	-70.2	130.5	1.598													
Tetrahydrofuran	-108	65	0.889	26.7	4.60 E-04			3	5	6	3	4	4	0	red	2
Tetrahydrothiophene	-96	119	1													
Tetralin	-35.8	206	0.973													2
Thioacetic Acid	-58	93	1.065													
Toluene	-95	111	0.87	27.9	5.60 E-04			6	3	4	4	10	7	3	0	2
Trichloroacetic Acid	58	197	1.629					3	6	6	10	6	7	0	0	
Trichloroacetonitrile	-42	83	1.44					5	6	6	7	10	0	0	red	

Trichloroethylene	-73	87.2	1.463	28.7	5.50 E-04											2

Table S2 COSMO-RS predicted solubility of paracetamol, acetanilide, and metacetamol at isolation temperature in the different solvents used for the solvent selection. Solubility calculated as gram of compound per 100 grams of solvent.

Solvent	COSMO-RS Calculated Solubility (g/100g)		
	**Paracetamol**	**Acetanilide**	**Metacetamol**
1,1,1-Trichloroethane	<0.01	1.77	0.01
1,1-Dichloroethene	<0.01	2.44	0.01
1,1-Diethoxypropane	0.06	1.16	0.12
1,2,3-Trimethoxypropane	0.96	5.56	1.89
1,2-Dichlorobenzene	<0.01	0.97	<0.01
1,2-Dichloroethane	0.03	7.76	0.06
1,2-Propanediol	11.03	13.85	16.36
1,3,5-Trimethylbenzene	<0.01	0.53	<0.01
1,3-Dimethyltetrahydropyrimidin-2(1H)-one	NA	30.35	NA
1,3-Dioxane	6.61	16.29	12.59
1,3-Propanediol	15.14	14.86	21.36
1,4-Dimethylbenzene	<0.01	0.86	<0.01
1,4-Dioxane	5.34	13.67	9.88
1,5-Pentanediol	8.31	11.21	12.28
1-Bromo-2-Chloroethane	0.02	4.81	0.03
1-Bromobutane	<0.01	1.27	0.01
1-Butanol	6.71	15.93	10.11
1-Chlorobutane	0.01	2.00	0.01
1-Hexanol	3.07	9.65	4.64
1-Methylnaphthalene	<0.01	0.80	<0.01
1-Octanol	1.62	6.26	2.48
1-Pentanol	4.49	12.29	6.86
1-Propanol	9.22	18.96	13.49
2,2,4-Trimethylpentane	<0.01	0.05	<0.01
2,2-Dimethoxypropane	0.10	1.61	0.19

2-(2-Hydroxyethoxy)Ethanol	14.80	16.43	19.91
2-[2-(2-Hydroxyethoxy)Ethoxy]Ethanol	11.08	14.79	17.16
2-Amino-1-Butanol	25.43	15.98	32.07
2-Bromobutane	0.01	1.60	0.01
2-Butanol	4.00	11.48	6.15
2-Butanone	10.49	17.48	18.88
2-Butoxyethanol	4.93	12.99	7.98
2-Ethoxy-2-Methylpropane	0.02	0.64	0.03
2-Ethoxyethanol	9.55	17.57	14.04
2-Ethylhexanol	1.49	7.17	2.27
2-Iodobutane	<0.01	0.86	0.01
2-Methoxy-2-Methylbutane	0.06	0.98	0.12
2-Methoxy-2-Methylpropane	0.19	2.13	0.36
2-Methoxyethanol	12.64	20.50	17.91
2-Methoxyethylether	1.04	6.27	1.95
2-Methyl-1-Propanol	6.83	17.49	10.35
2-Methylbutan-2-ol	2.85	10.78	4.41
2-Methylpentane	<0.01	0.06	<0.01
2-Methyltetrahydrofuran	3.67	11.68	7.87
2-Octanol	1.13	4.89	1.75
2-Pentanol	2.30	8.03	3.48
2-Pentanone	4.47	12.24	9.74
2-Phenylethanol	3.26	11.94	5.12
2-propanethiol	0.02	4.87	0.04
2-Propanol	5.54	13.58	8.13
3-Fluorotoluene	<0.01	0.79	<0.01
3-Methyl Thiophene	<0.01	1.93	0.01
3-Methyl-1-Butanol	4.60	12.39	6.90
3-Pentanone	0.99	6.92	2.10
4-Fluorotoluene	<0.01	0.79	<0.01
4-Methyl-1-Pentanol	4.01	11.35	6.16
5-Methyl furfural	9.23	13.17	16.21

Acetone	18.74	23.64	28.21
Acetonitrile	5.56	13.28	11.22
Acetyl Acetate	0.60	4.61	1.27
Aniline	3.35	17.01	6.15
Anisole	0.02	2.01	0.04
Benzene	0.01	3.62	0.02
Benzylalcohol	3.98	14.59	6.18
Bromobenzene	<0.01	0.88	<0.01
Bromoform	<0.01	2.36	0.01
Butyl Acetate	1.03	6.73	2.31
Butyl Lactate	4.09	10.56	7.83
Butyric Acid	4.38	27.52	5.66
Butyronitrile	3.45	13.17	7.79
Carbon Tetrachloride	<0.01	0.15	<0.01
Chlorobenzene	<0.01	1.24	0.01
Chloroform	0.01	10.58	0.02
cis-Decalin	<0.01	0.04	<0.01
Cumene	<0.01	0.61	<0.01
Cyclohexane	<0.01	0.05	<0.01
Cyclohexanone	13.06	18.90	20.48
Cyclopentane	<0.01	0.08	<0.01
Cyclopentanone	14.40	22.10	22.07
Cyclopentyl Methyl Ether	0.05	1.04	0.09
Dibutylether	0.01	0.31	0.02
Dichloromethane	0.06	19.20	0.11
Diethylcarbonate	0.90	6.94	1.88
Diethylene Glycol Butyl Ether	3.97	9.93	6.63
Diethylether	0.14	2.27	0.27
Diethylsulfide	0.01	2.17	0.02
Dimethoxyethane	0.42	6.78	0.79
Dimethoxymethane	1.00	8.12	2.30
Dimethyl Carbonate	1.32	8.37	2.84

Dimethyl Disulfide	0.01	3.10	0.02
Dimethyl Sulfide	0.04	8.78	0.07
Dodecane	<0.01	0.03	<0.01
Ethanethiol	0.04	9.90	0.08
Ethanol	13.37	20.69	18.64
Ethyl Acetate	8.97	17.36	15.41
Ethyl Formate	7.30	18.31	13.53
Ethyl Isovalerate	0.21	2.90	0.42
Ethyl Lactate	7.35	14.80	12.46
Ethyl Phenyl Ether	0.01	1.15	0.02
Ethyl Propanoate	1.22	8.10	2.71
Ethylene Glycol	8.21	9.40	13.31
Fluorobenzene	<0.01	1.34	0.01
Formamide	9.23	3.33	14.96
Furfural	6.46	11.62	11.67
Furfuryl Alcohol	5.05	16.00	7.70
Glycerol Carbonate	2.01	5.24	3.24
Heptane	<0.01	0.04	<0.01
Hexane	<0.01	0.05	<0.01
Iodobenzene	<0.01	0.60	<0.01
Iodomethane	0.01	5.21	0.02
Isoamyl Acetate	1.15	6.14	2.59
Isobutyl Acetate	0.81	5.95	1.78
Isopropyl Acetate	2.65	10.08	5.94
Isopropyl butadecanoate	0.01	0.35	0.03
Isopropyl Ether	0.02	0.70	0.04
Limonene	<0.01	0.23	<0.01
Methanedithione	<0.01	0.19	<0.01
Methanoic Acid	16.46	54.38	18.21
Methanol	28.49	36.00	35.74
Methyl Acetate	7.68	17.13	14.01
Methyl Isobutyl Ketone	3.31	10.20	7.56

Methyl Lactate	7.09	14.56	11.73
Methylbutyl Ketone	2.95	9.48	6.67
Methylcyclohexane	<0.01	0.05	<0.01
Methylcyclopentane	<0.01	0.06	<0.01
Methylisopropyl Ketone	6.45	15.18	12.98
N,N-Dimethylacetamide	NA	39.34	NA
N,N-Dimethylaniline	0.01	1.21	0.01
N,N-Dimethylformamide	NA	43.47	NA
N-Methyl-2-Pyrrolidone	NA	33.92	NA
N-Methylformamide	33.09	27.31	39.26
Nitrobenzene	0.10	2.83	0.20
Nitromethane	0.17	2.72	0.35
Octyl Acetate	0.25	2.26	0.54
Pentane	<0.01	0.06	<0.01
Pentylacetate	0.82	5.35	1.84
Perfluoro-2-butyltetrahydrofuran	<0.01	<0.01	<0.01
Perfluorohexane	<0.01	<0.01	<0.01
Perfluorotoluene	<0.01	0.03	<0.01
Piperidine	47.60	35.62	35.24
Propanenitrile	6.71	17.57	13.38
Propionic Acid	4.94	29.94	6.15
Propyl Acetate	4.08	12.34	8.52
Propylene Carbonate	1.04	3.39	2.16
Pyridine	NA	42.18	NA
Tert-Butyl Acetate	2.50	8.43	5.76
Tetrachloroethene	0.01	4.05	0.01
Tetrahydrofuran	22.70	28.21	31.79
Tetrahydrothiophene	0.02	3.97	0.04
Tetralin	<0.01	0.44	<0.01
Thioacetic Acid	1.06	11.65	2.05
Toluene	<0.01	1.50	0.01
Trichloroacetonitrile	<0.01	0.42	0.01

Trichloroethylene	<0.01	1.67	<0.01
Triethylamine	<0.01	0.14	<0.01
Trifluoroacetic Acid	NA	NA	28.51
Trifluoroethanol	2.66	26.76	3.32
Trifluorotoluene	<0.01	0.44	<0.01
Water	0.87	0.28	1.20

Table S3 Slurry (suspension) mass fraction of the experiments N1, N3, and N12 reported by Ottoboni et al. (2020)[31] used for the filtration and washing models validation stage.

**Compound in suspension**	**N1**	**N3**	**N12**
Crystallisation solvent	0.7659	0.8065	0.7527
Paracetamol	0.2256	0.1864	0.2383
Acetanilide	0.0040	0.0033	0.0043
Metacetamol	0.0045	0.0037	0.0048
Wash solvent	0.0000	0.0000	0.0000

Table S4 Mother liquor mass fraction of the experiments N1, N3, and N12 reported by Ottoboni et al. (2020)[31] used for the filtration and washing models validation stage.

**Compound in mother liquor**	**N1**	**N3**	**N12**
Crystallisation solvent	0.8653	0.9175	0.9416
Paracetamol	0.1250	0.0745	0.0477
Acetanilide	0.0046	0.0038	0.0051
Metacetamol	0.0051	0.0042	0.0057
Wash solvent	0.0000	0.0000	0.0000

Table S5 Solvent properties used in model B to simulate filtration and washing process[31].

	**Molecular weight (g/mol)**	**Density (kg/m^3)**	**Heat capacity (J/mol K)**	**Dynamic viscosity (Pa s)**
Paracetamol	151.16	1260	280	-
Acetanilide	135.16	1220	280	-
Metacetamol	151.16	1249	280	-

Ethanol	46.07	789	37	1.204E-03
2-propanol	60.1	786	150	1.96E-03
3-methyl-1-butanol	88.15	810	190	4.37E-03
n-heptane	100.2	684	210	-
n-dodecane	170.33	750	380	-

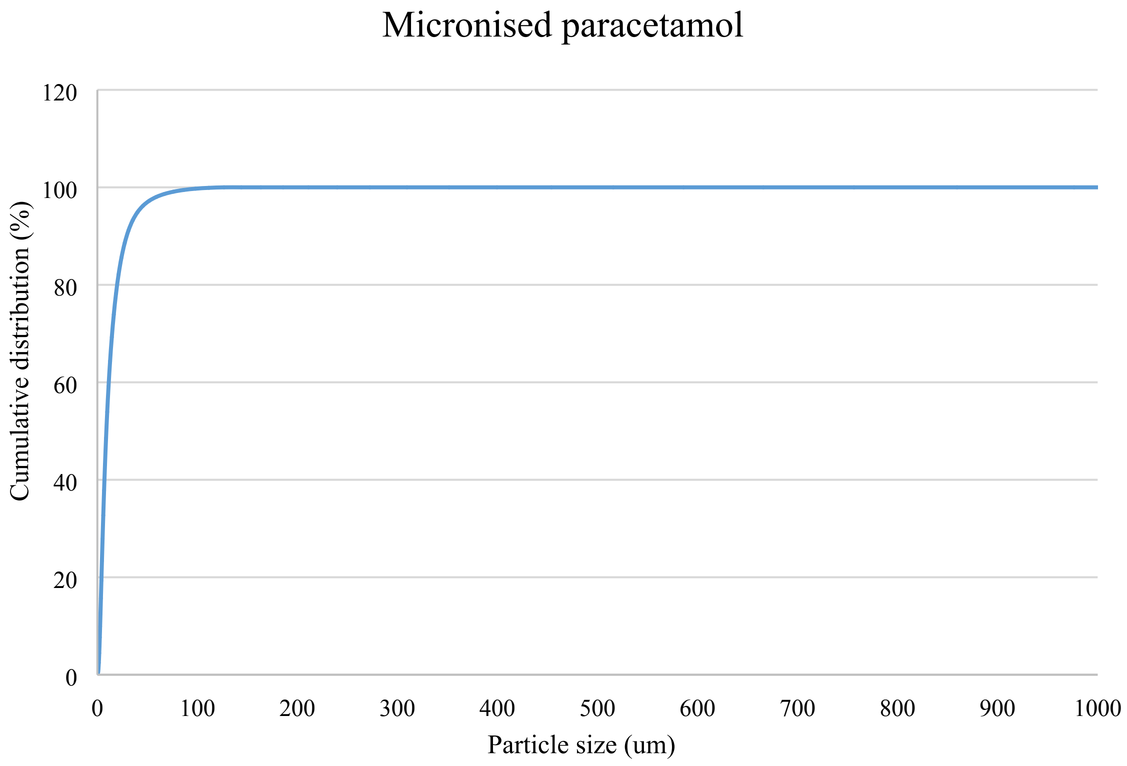

Figure S1 Cumulative distribution of the particle size analysis of micronized paracetamol.

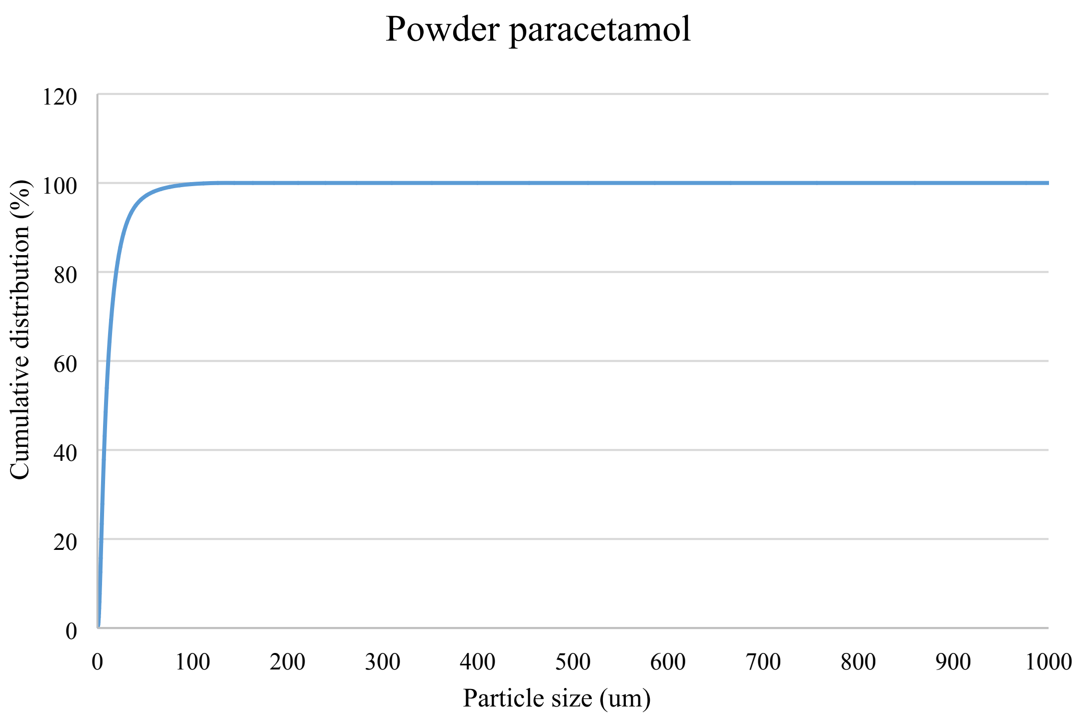

Figure S2 Cumulative distribution of the particle size analysis of powder paracetamol.

Table S6 Mean volumetric particle size and standard deviation of the particles size distribution of the micronized and powder paracetamol used for the filtration and washing model B used for the prediction of isolation performances.

	**Micronised**	**Powder**
D50	13.85	77.36
Standard deviation	16.16	174.43

Table S7 Paracetamol, acetanilide, and metacetamol COSMOtherm solubility prediction in ethanol, 2-propanol, 3-methyl-1-butanol, n-heptane, and n-dodecane at isolation temperature (25°C).

Solvent	Paracetamol solubility (g/g at 25°C)	Acetanilide solubility (g/g at 25°C)	Metacetamol solubility (g/g at 25°C)
Ethanol	0.1445	0.2256	0.2003
2-propanol	0.0812	0.1508	0.0905
3-methyl-1-butanol	0.0506	0.1365	0.0761
n-heptane	7.694E-07	5.676E-04	1.8458E-06
n-dodecane	4.776E-07	3.415E-04	1.1513E-06

Table S8 Paracetamol, acetanilide, and metacetamol solubility in ethanol, 2-propanol, 3-methyl-1-butanol, n-heptane, and n-dodecane at isolation temperature (25°C) experimentally measured with gravimetric method.

Solvent	Paracetamol solubility (g/g at 25°C)	Acetanilide solubility (g/g at 25°C)	Metacetamol solubility (g/g at 25°C)
Ethanol	0.2056	0.3322	0.2944
2-propanol	0.1141	0.1957	0.1948
3-methyl-1-butanol	0.0549	0.1656	0.1049
n-heptane	0.0003	5.676E-04	0.0004
n-dodecane	0.0007	0.0019	0.0011

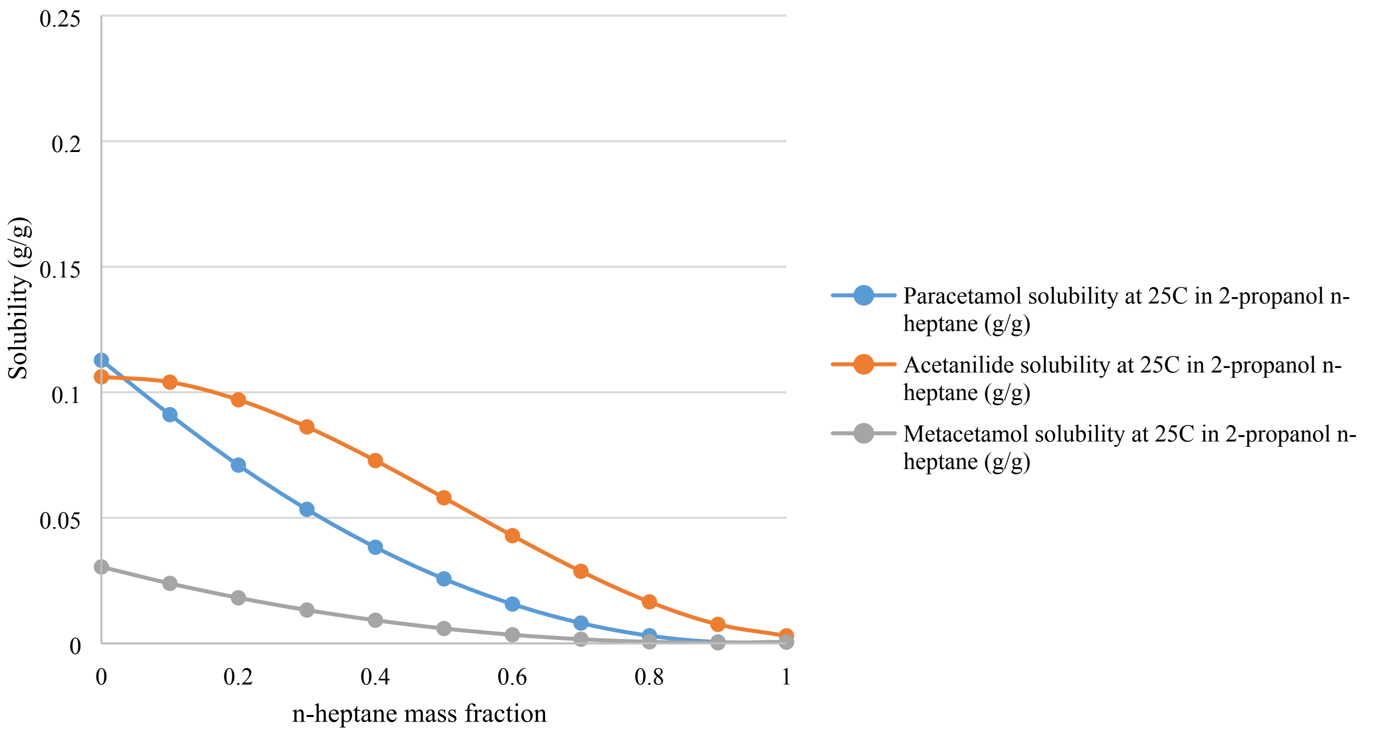

Figure S3 COSMOtherm binary plot solubility of paracetamol (API), acetanilide, and metacetamol in a gradient crystallisation solvent (2-propanol) and wash solvent mixture (n-heptane).

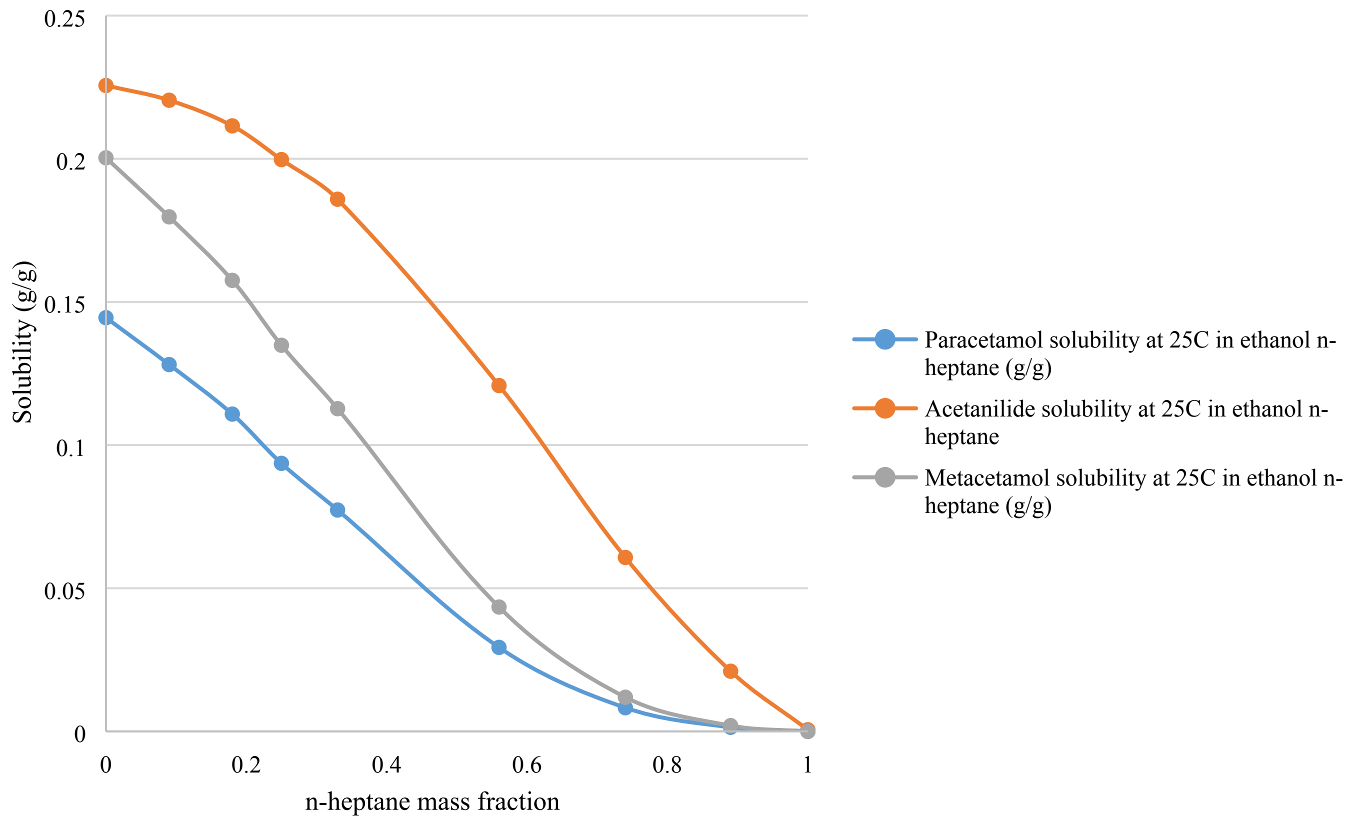

Figure S4 COSMOtherm binary plot solubility of paracetamol (API), acetanilide, and metacetamol in a gradient crystallisation solvent (ethanol) and wash solvent mixture (n-heptane).

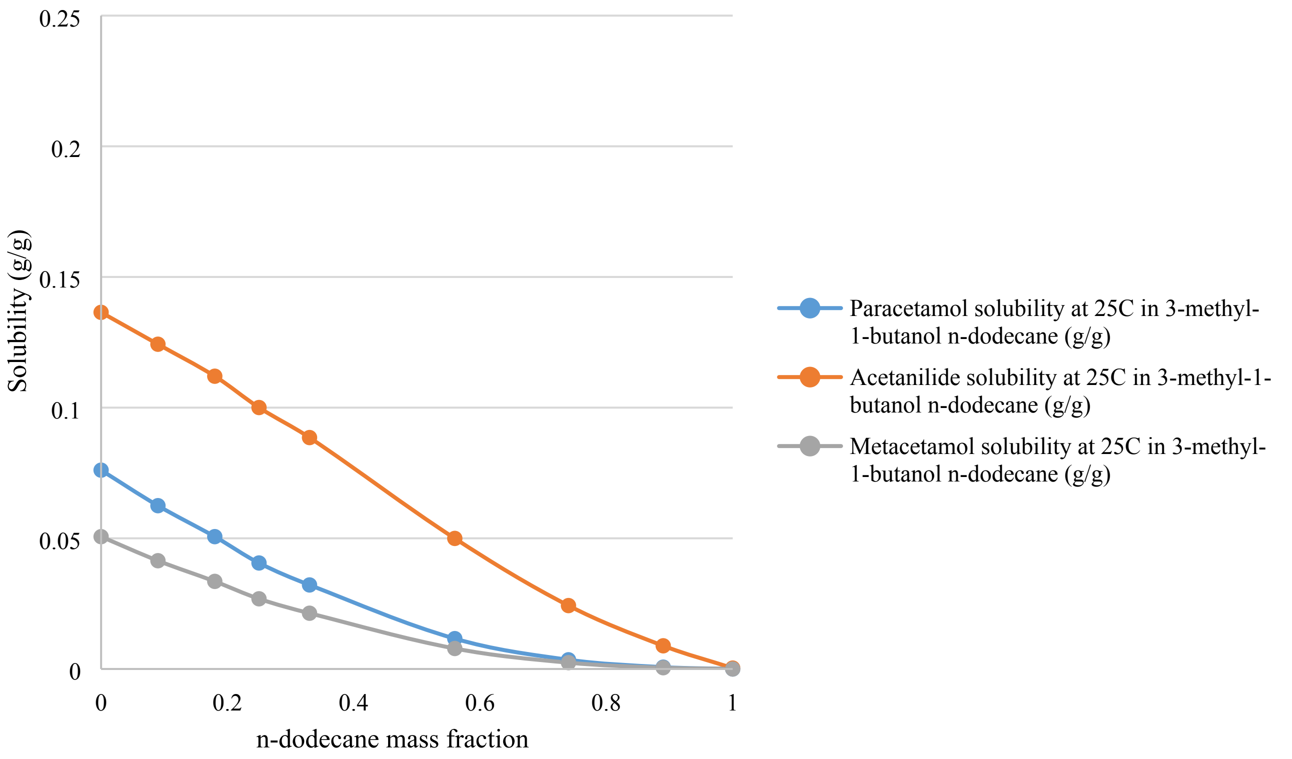

Figure S5 COSMOtherm binary plot solubility of paracetamol (API), acetanilide, and metacetamol in a gradient crystallisation solvent (3-methyl-1-butanol) and wash solvent mixture (n-dodecane).

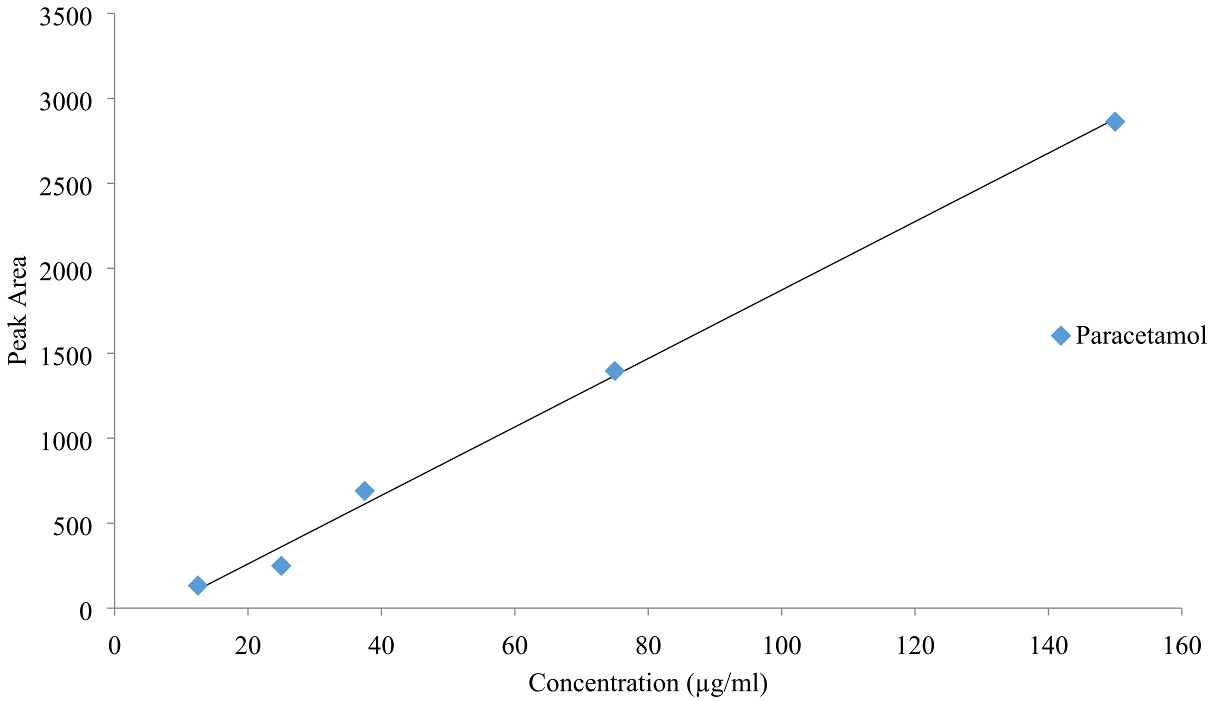

Figure S6 HPLC calibration curve of paracetamol.

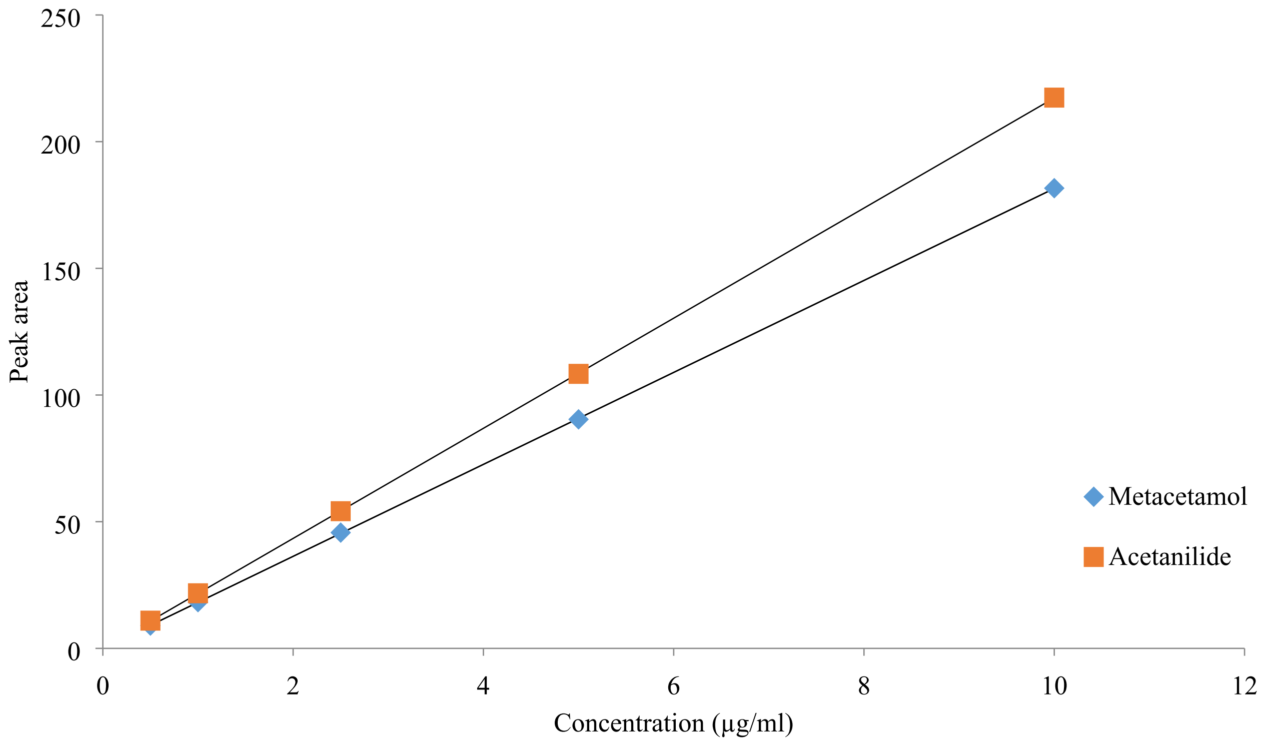

Figure S7 HPLC calibration curve of metacetamol and acetanilide.

Table S9 HPLC peak areas and concentration of paracetamol, acetanilide, and metacetamol dissolved in the filtrate sample collected after wash 1 and wash 2 of experiments N1, N3, and N12 of the DoE reported by Ottoboni at el. (2020)[31].

	**N1**	**N3**	**N12**
Peak area paracetamol filtrate after wash 1 (mAu*s)	17834.80000	6770.44873	6084.40967

Peak area acetanilide filtrate after wash 1 (mAu*s)	613.29498	292.67703	351.77359
Peak area metacetamol filtrate after wash 1 (mAu*s)	682.62720	362.82321	399.57913
Peak area paracetamol filtrate after wash 2 (mAu*s)	3917.10645	6744.52100	1548.12317
Peak area acetanilide filtrate after wash 2 (mAu*s)	45.81313	238.04901	55.21097
Peak area metacetamol filtrate after wash 2 (mAu*s)	52.92332	247.35185	62.14240
Concentration paracetamol filtrate after wash 1 (µg/mL)	164877.2175	85421.0364	56248.4882
Concentration paracetamol filtrate after wash 1 (µg/mL)	5786.6242	2901.0743	2685.5984
Concentration paracetamol filtrate after wash 1 (µg/mL)	6310.6776	3788.8060	3576.8741
Concentration paracetamol filtrate after wash 2 (µg/mL)	36212.4393	62351.0135	14311.9205
Concentration paracetamol filtrate after wash 2 (µg/mL)	432.2608	1817.3736	421.5055
Concentration paracetamol filtrate after wash 2 (µg/mL)	489.2597	2214.1958	556.2742

Table S10 Predicted filtration and washing parameter obtained from model B.

	N1	N3	N12
Cake height after filtration (m)	0.0305	0.0314	0.0332
Mass liquid left in the cake after filtration (kg)	0.00473	0.00476	0.0051
Filtration time first dose (s)	31.151	6.584	205.346
Filtration time second dose (s)	79.818	15.991	-
Volumetric flow rate of filtrate at the end of filtration (m^3/s)	5.3542E-07	3.4030E-06	1.5144E-07
Washing time total (s)	40.525	3.12	74.09

Table S11 Simulated concentration of paracetamol, acetanilide, and metacetamol removed during wash 1, 2, and 3 (collected in the filtrate phase) of isolation optimal strategy case 3. A filtration and washing process was simulated and a powder grade paracetamol; the cake was washed using 3 washes, using each time 1.76 equivalent cake volume of wash solvent (equivalents to 4 cake void volume). Wash 1 was done with a solvent mixture of 50-50% of ethanol and n-heptane to prevent anti-solvent effect as reported by Ottoboni et al. (2020)[31]. Wash 2 and 3 were done using 2 aliquots of pure n-heptane with 0.88 equivalent cake volumes of solvent. Purity achieved corresponds to 98.45%.

Simulated case 3	Concentration in filtrate collected after filtration (g/g filtrate)	Concentration in filtrate collected after W1 (g/g filtrate)	Concentration in filtrate collected after W2 (g/g filtrate)	Concentration in filtrate collected after W3 (g/g filtrate)
Paracetamol	0.1250	0.6219	0.3616	0.2123
Acetanilide	0.0046	0.0227	0.0132	0.0077
Metacetamol	0.0051	0.0254	0.0147	0.0087
Yield (%)	68.97	64.17	59.61	55.27

Table S12 Simulated concentration of paracetamol, acetanilide, and metacetamol removed during wash 1, 2, and 3 (collected in the filtrate phase) of isolation optimal strategy case 4. A filtration and washing process was simulated and a micronised grade paracetamol; the cake was washed using 3 washes, using each time equivalent cake volume of wash solvent (equivalents to 4 cake void volume). Wash 1 was done with a solvent mixture of 50-50% of ethanol and n-heptane to prevent anti-solvent effect as reported by Ottoboni et al. (2020)[31]. Wash 2 and 3 were done using 2 aliquots of pure n-heptane with 1.38 equivalent cake volumes of solvent. Purity achieved corresponds to 98.51%.

Simulated case 4	Concentration in filtrate collected after filtration (g/g filtrate)	Concentration in filtrate collected after W1 (g/g filtrate)	Concentration in filtrate collected after W2 (g/g filtrate)	Concentration in filtrate collected after W3 (g/g filtrate)
Paracetamol	0.1250	0.6500	0.3618	0.2034

Acetanilide	0.0046	0.0237	0.0132	0.0074
Metacetamol	0.0051	0.0265	0.0147	0.0083
Yield (%)	57.36	52.41	48.78	57.36
